# Molecular Links Between Smoking, COPD, and Lung Cancer: A DNA Methylation Perspective

**DOI:** 10.3390/cancers18081273

**Published:** 2026-04-17

**Authors:** Camila Bernal Forigua, Litzy Gisella Bermúdez, Alejandra Cañas Arboleda, Rafael R. Ariza, Maria Teresa Roldán, Maria Teresa Morales, Daniel Mauricio González Cubides, Adriana Rojas

**Affiliations:** 1 Institute of Human Genetics, School of Medicine, Pontificia Universidad Javeriana, Carrera 7 No. 40-62, Edificio 32, Bogotá 110231, Colombia; camila-bernal@javeriana.edu.co (C.B.F.); go.daniel@javeriana.edu.co (D.M.G.C.); 2 Department of Genetics, University of Córdoba, 14071 Córdoba, Spain; 3 Departamento de Medicina Interna, School of Medicine, Pontificia Universidad Javeriana, Carrera 7 No. 40-62, 2.Ed. Fernando Barón, Bogotá 110231, Colombia; 4 Maimónides Biomedical Research Institute of Córdoba (IMIBIC), 14004 Córdoba, Spain; 5 Reina Sofía University Hospital, 14004 Córdoba, Spain

**Keywords:** epigenetics, DNA methylation, lung cancer, chronic obstructive pulmonary disease, smoking

## Abstract

**Background:** DNA methylation alterations represent a key epigenetic mechanism linking environmental exposures to disease pathogenesis. The present study aimed to identify differentially methylated genes and shared biological processes associated with lung cancer (LuCa), chronic obstructive pulmonary disease (COPD) and tobacco exposure. **Methods:** A comprehensive literature search was performed in PubMed to identify studies evaluating DNA methylation in LuCa, COPD and smoking-related models. A total of 117 articles were selected, including 83 studies on lung cancer, 18 on COPD and 16 on smoking exposure. Genes exhibiting statistically significant methylation changes relative to controls were extracted from each study. To provide additional support for these findings, differential methylation signatures were further evaluated using The Cancer Genome Atlas (TCGA) lung adenocarcinoma (LUAD) and lung squamous cell carcinoma (LUSC) datasets. Functional and transcription factor motif enrichment analyses were subsequently conducted to identify shared biological pathways and regulatory mechanisms. **Results:** In total, 324 genes displaying altered methylation patterns across these conditions were identified. Seven tumor suppressor genes (*CDKN2A*, *CDH13*, *MGMT*, *MIR137*, *DAPK1*, *RARB*, and *RASSF1A*) consistently exhibited hypermethylation in both lung cancer and in association with smoking exposure. In addition, *AHRR* hypomethylation emerged as a shared epigenetic hallmark across all three conditions. TCGA-based analyses confirmed several of these methylation patterns and revealed subtype-specific methylation profiles associated with smoking history. Functional enrichment highlighted common biological processes and signaling pathways, particularly those related to transcriptional regulation, apoptosis and cancer-associated pathways. **Conclusions:** These results provide an integrative overview of shared DNA methylation alterations associated with smoking exposure, COPD, and lung cancer, and suggest potential DNA methylation candidates that may be relevant for future biomarker development and mechanistic studies.

## Simple Summary

DNA methylation alterations represent a key epigenetic mechanism linking environmental exposures to disease pathogenesis. This study aimed to identify differentially methylated genes and shared biological processes associated with lung cancer (LuCa), chronic obstructive pulmonary disease (COPD), and tobacco exposure. A comprehensive literature search in PubMed identified 117 relevant studies (83 on lung cancer, 18 on COPD, and 16 on smoking exposure). In total, 324 differentially methylated genes were identified. Seven tumor suppressor genes (*CDKN2A, CDH13, MGMT, MIR137, DAPK1, RARB*, and *RASSF1A*) were consistently hypermethylated in both lung cancer and smoking exposure, while *AHRR* hypomethylation was common across all three conditions. These findings were validated using The Cancer Genome Atlas (TCGA) datasets for lung adenocarcinoma (LUAD) and lung squamous cell carcinoma (LUSC). Enrichment analysis revealed that differentially methylated genes were associated with cell cycle regulation, MAPK, and estrogen signaling pathways, and showed overrepresentation of binding motifs for the transcription factors MAZ and AP-2α. Overall, these results support the role of epigenetic alterations as a shared molecular layer linking tobacco exposure, COPD, and lung carcinogenesis, and highlight their potential for biomarker development in risk assessment and early detection.

## 1. Introduction

Cancer encompasses a group of complex diseases characterized by aberrant and uncontrolled cellular proliferation, which results from the evasion of endogenous control mechanisms. This process involves genetic and epigenetic alterations that modify gene expression, thereby promoting the acquisition of phenotypic characteristics that facilitate tumor survival and progression [1].

Among these pathologies, lung cancer (LuCa) stands out as the most common neoplasm and the leading cause of cancer-related mortality worldwide, with more than 2.4 million registered cases and 1.8 million annual deaths [2,3]. Survival in lung cancer depends on three critical factors: disease stage, functional status, and the molecular characteristics of the tumor. Significant advances in molecular diagnostics and targeted therapies have substantially improved survival rates. In part, this progress has been driven by the incorporation of liquid biopsy as an alternative to conventional tumor tissue biopsies, providing a novel approach for lung cancer prevention, early detection, and accurate molecular profiling. Recent bibliometric analyses have highlighted the growing relevance of circulating tumor DNA (ctDNA) for the identification of molecular and epigenetic alterations with potential diagnostic value in lung cancer [4].

The development of LuCa is influenced by both environmental and genetic factors. Key environmental risk factors include exposure to carcinogens (radon, asbestos, arsenic, among others), air pollution, wood smoke, and, particularly, tobacco smoking [5]. Tobacco consumption remains the leading preventable cause of death worldwide, accounting for 8.7 million deaths in 2019 according to the World Health Organization [6]. Beyond tobacco exposure, environmental pollutants such as fine particulate matter (PM2.5) have been associated with molecular and epigenetic alterations involved in lung carcinogenesis. Notably, these alterations include changes in DNA methylation patterns, covalent histone modifications, and dysregulation of non-coding RNAs, highlighting the complex epigenetic response to environmental exposures [7,8].

In addition to being associated with numerous chronic diseases, smoking has been established as a determinant factor in the etiology of lung cancer and Chronic Obstructive Pulmonary Disease (COPD), the latter being the respiratory condition with the highest incidence rates and a significant risk factor for lung cancer development [9].

COPD, which is characterized by progressive airflow limitation, shares multiple pathogenic mechanisms with LuCa, including chronic inflammation, alterations in cell cycle regulation, and proteinase secretion by immune and stromal cells. It has also been proposed that both genetic and epigenetic changes may contribute to the simultaneous development of both diseases [10,11,12].

In this context, epigenetics emerges as a crucial component in LuCa, functioning as a link between alterations in the cellular microenvironment and changes in gene expression that promote tumor initiation and progression [13,14]. Among epigenetic mechanisms, DNA methylation stands out as a fundamental process in the regulation of gene expression. This process involves the covalent transfer of a methyl group to the carbon 5 of cytosine, forming 5-methylcytosine (5mC) [15]. 5mC is primarily localized in dinucleotides known as CpG sites, which are concentrated in regions called “CpG islands”. The methylation status of these CpG islands is crucial for gene regulation. When CpG islands are in promoter regions, a hypermethylated state is generally associated with transcriptional repression, while loss of methylation is linked to gene activation. Tumor cells frequently exhibit aberrant methylation patterns, characterized by global hypomethylation alongside specific promoter hypermethylation, which generally corresponds to tumor suppressor genes [16].

It has been documented that smoking induces changes in DNA methylation patterns, which may partially explain its impact on lung cancer risk [17]. Studies have shown that COPD patients exhibit distinct DNA methylation patterns compared to individuals without the disease, which may contribute to an increased risk of developing LuCa. Specifically, elevated levels of DNA methyltransferases, such as DNMT1, have been associated with hypermethylation of tumor suppressor gene promoters in lung cancer patients, particularly those with COPD [18]. Furthermore, studies have shown that methylation profiles in lung tumors from COPD patients reveal a higher degree of methylation and gene repression compared to non-COPD lung cancer patients, suggesting that the epigenetic landscape is altered in a manner that promotes tumorigenesis in the context of COPD [19]. These observations support the hypothesis that epigenetic alterations may represent a molecular link between COPD and lung cancer development.

This study aims to identify differentially methylated genes and dysregulated biological processes common to lung cancer and its risk factors, such as COPD and smoking. To achieve this, we searched the PubMed database for articles identifying differentially methylated genes in models of LuCa, COPD and smoking history. The identified genes were subsequently integrated with functional enrichment analyses to explore biological processes and signaling pathways potentially affected by DNA methylation alterations across these conditions and to provide an integrative overview of shared epigenetic patterns reported in the literature.

## 2. Materials and Methods

### 2.1. Search Strategy, Inclusion Criteria and Data Collection

A comprehensive literature search was conducted in the PubMed database to identify studies evaluating DNA methylation alterations in LuCa, COPD, and smoking-related human models. The search was carried out independently for each condition using the following search terms: “DNA METHYLATION”, “LUNG CANCER”, “COPD” and “SMOKERS”. Studies were screened based on title, abstract, and full-text evaluation according to the eligibility criteria defined below. The aim of this approach was to obtain a curated literature-based dataset of genes reported as differentially methylated in each condition, which was subsequently integrated with The Cancer Genome Atlas (TCGA) validation and functional enrichment analyses.

**Figure 1:**
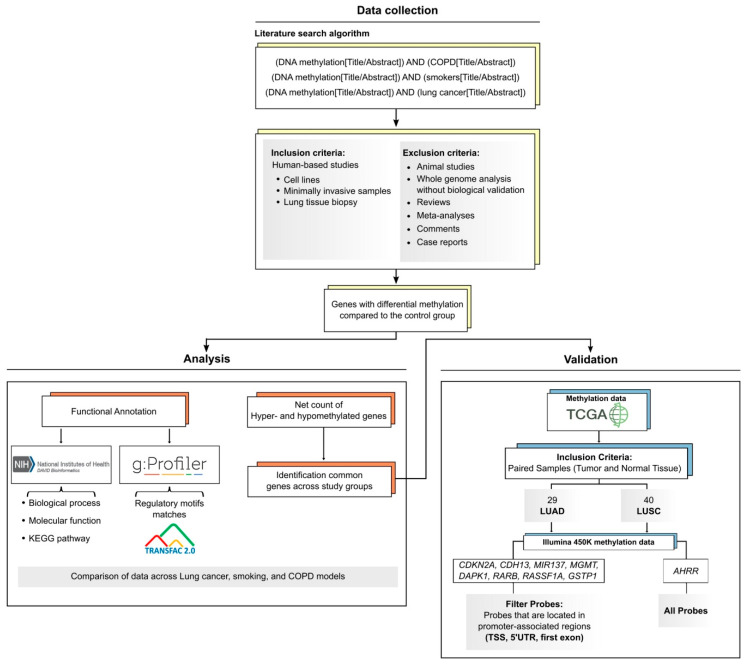
Schematic flow chart depicting the literature search strategy and subsequent analysis pipeline. A search for human evidence of aberrant gene methylation in LuCa, COPD and smokers was conducted. The resulting list of genes was compared among conditions and functionally annotated for further analysis.

Studies that focused on human models, including cell lines, minimally invasive clinical samples and lung tissue biopsies, were included in this work. In contrast, studies based on animal models, genome-wide methylation studies without biological validation, as well as review articles, meta-analyses and case reports, were excluded (Figure 1).

Following the comprehensive search criteria, a total of 220 research articles were initially identified: 177 articles related to lung cancer, 19 focused on smokers, and 24 addressing COPD. After rigorously applying inclusion and exclusion criteria, the final sample comprised 83 LuCa studies, 16 studies on smokers, and 18 COPD research models (Table S1).

We then extracted from the eligible articles a list of genes with statistically significant changes in DNA methylation levels, either hypermethylation or hypomethylation, compared to the control group in each study. Differential methylation was considered when studies reported statistically significant differences according to their original statistical analyses (*p* < 0.05). The identified genes were classified based on their association with LuCa, COPD, or smoking. After compiling the lists of methylated genes for each group, duplicates across studies were removed, leaving a single record for each gene (Figure 1, Table S2).

### 2.2. Functional Annotation Analysis

To identify the Gene Ontology (GO) biological processes (BP), molecular functions (MF) [20,21], as well as the signaling pathways (KEGG pathways) [22,23] associated with the differentially methylated genes identified in LuCa, COPD, and smoking models, a functional enrichment analysis was performed using the Database for Annotation, Visualization and Integrated Discovery (DAVID- v2025_2) tool [24] (Figure 1).

In addition, an overrepresentation analysis (ORA) was conducted using the g: GOSt function from the g:Profiler tool (version e113_eg59_p19_6be52918) [25,26,27] to identify significant matches with regulatory motifs from the TRANSFAC database. This analysis was used to explore potential transcription factor binding motifs associated with the identified methylated genes (Figure 1).

### 2.3. Analysis of DNA Methylation and Gene Expression Profiles in TCGA Lung Cancer Datasets

DNA methylation (Illumina HumanMethylation450K, preprocessed with SeSAMe v1.22.2 [28]) and gene expression (TPM values from the STAR-Counts workflow) data for Lung Adenocarcinoma (LUAD) and Lung Squamous Cell Carcinoma (LUSC) were retrieved from The Cancer Genome Atlas (TCGA) via TCGAbiolinks v2.34 [29,30,31,32]. Only patients with matched tumor and adjacent normal tissue samples were retained, yielding 29 and 40 pairs for methylation analysis and 58 and 51 pairs for expression analysis in LUAD and LUSC, respectively.

For methylation analysis, probe selection was performed using the IlluminaHumanMethylation450kanno.ilmn12.hg19 v0.6.1 annotation package [33]. Probes were filtered to retain those mapping to promoter-associated regions (TSS200, TSS1500, 5′UTR, and first exon) for nine target genes (*CDKN2A*, *CDH13*, *MIR137*, *MGMT*, *DAPK1*, *RARB*, *RASSF1A*, *GSTP1*, and *AHRR*). Probe pairs containing missing values were excluded pairwise (Figure 1). All promoter-associated probes per gene were aggregated and tested jointly. Differential methylation between matched tumor and adjacent normal tissues was assessed using paired Wilcoxon signed-rank tests. Associations between methylation levels and smoking pack-years were evaluated using Spearman correlation, while associations with smoking categories were assessed using Kruskal–Wallis tests followed by post hoc Dunn’s tests [34]. Smoking categories were defined according to TCGA clinical annotation as follows: lifelong non-smokers (category 1; <100 cigarettes in lifetime), current reformed smokers for >15 years (category 2), current reformed smokers for ≤15 years (category 3), and current smokers (category 4).

Gene expression analysis was specifically conducted for *MAZ* and *TFAP2A* to evaluate whether the transcription factors identified in the motif overrepresentation analysis showed altered expression in lung cancer. Differential expression between matched tumor and adjacent normal tissues was likewise assessed using paired Wilcoxon signed-rank tests on log-transformed TPM values.

For both methylation and expression analyses, *p*-values were adjusted using the Benjamini–Hochberg false discovery rate (FDR) method, with statistical significance defined as FDR < 0.05. All analyses were conducted in R v4.4.1 [35] R using dplyr [36] and ggplot2 [37].

## 3. Results

### 3.1. Identification of Genes with Aberrant Methylation Profiles in Lung Cancer, Cigarette Smoke Exposure and COPD

**Figure 2:**
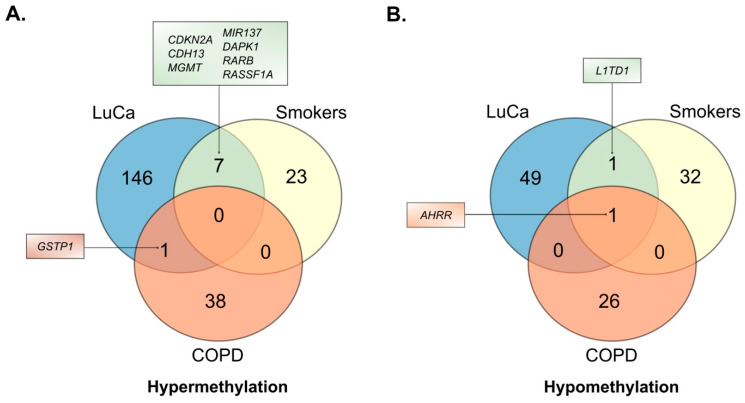
Comparison of differentially methylated genes in the LuCa, COPD and smoking study models. (**A**) Venn diagram showing the distribution of hypermethylated genes identified in lung cancer (LuCa), smokers, and COPD datasets. Seven genes (*CDKN2A, CDH13, MGMT, MIR137, DAPK1, RARB,* and *RASSF1A*) were shared between lung cancer and smokers, whereas *GSTP1* was common to lung cancer and COPD. (**B**) Venn diagram showing the overlap of hypomethylated genes across the same conditions. *L1TD1* was shared between lung cancer and smokers, while *AHRR* was common across all three conditions. Numbers indicate the total genes uniquely or jointly methylated in each condition. Abbreviations: LuCa, lung cancer; COPD, chronic obstructive pulmonary disease.

A comprehensive analysis revealed a total of 324 genes with distinct methylation pattern alterations. In LuCa models, 154 genes predominantly displayed hypermethylation, while 51 exhibited hypomethylation. In models of cigarette smoke exposure, 30 hypermethylated and 34 hypomethylated genes were identified. For COPD study models, DNA methylation changes were observed in 39 hypermethylated and 27 hypomethylated genes (Figure 2).

After generating the list of differentially methylated genes, comparative analyses were performed to identify common genes across study groups. Seven hypermethylated genes were identified as shared between lung cancer and cigarette smoke exposure groups. These genes have been previously reported as tumor suppressors whose expression can be regulated by DNA methylation [38,39,40,41,42,43,44]. The identified genes were *Cyclin Dependent Kinase Inhibitor 2A* (*CDKN2A), Cadherin 13 (CDH13), O-6-Methylguanine-DNA Methyltransferase (MGMT), MicroRNA 137 (MIR137), Death Associated Protein Kinase 1 (DAPK1), Retinoic Acid Receptor Beta (RARB), and Ras Association Domain Family Member 1 (RASSF1A)* (Figure 2A) [38,39,40,41,42,43,44]. *Glutathione S-transferase Pi 1 (GSTP1)* was identified as a hypermethylated gene common to LuCa and COPD. This gene encodes an isoenzyme primarily involved in the pulmonary metabolism of xenobiotics derived from tobacco smoke [45]. Furthermore, no common hypermethylated genes were identified between COPD and smokers, and no genes were shared across all three conditions (LuCa, COPD, and smokers) (Figure 2A).

Analysis of common hypomethylated genes revealed the loss of methylation in the *LINE1 type transposase domain containing 1* (*L1TD1*) gene in both the LuCa and smoking groups (Figure 2B). *LINE1* methylation is widely used as a surrogate indicator of global DNA methylation levels [46]. Hypomethylation of repetitive elements, such as *LINE1*, has been linked to genomic instability and has been demonstrated to serve as a prognostic marker in various malignancies [46].

Interestingly, hypomethylation of the *Aryl Hydrocarbon Receptor Repressor (AHRR)* gene was identified across all three studied conditions: LuCa, smoking, and COPD (Figure 2B). DNA methylation alterations in the *AHRR* gene have been widely reported as markers of smoking exposure, with significant correlations observed between *AHRR* hypomethylation and lung function parameters, cigarette consumption levels, and nicotine dependence. Specifically, hypomethylation at the cg05575921 site within the *AHRR* gene has been consistently reported in smoking-related studies [47].

Considering that epigenetic changes are tissue-specific, the sample types in which DNA methylation changes of common genes had been reported were compared. The comparison is detailed in Table 1. The studies analyzed revealed that changes in *AHRR* gene methylation patterns were identified in whole blood samples for LuCa and smokers. For hypermethylated genes, many of these changes were observed in lung epithelial samples, such as lung biopsies (including tumor tissues) and bronchial brushing cells. Genes such as *GSTP1, CDKN2A, MGMT, DAPK1,* and *RASSF1A* showed methylation changes in lung epithelial cells. Notably, hypermethylation of the *RASSF1A* gene has also been reported in minimally invasive sputum samples from both LuCa patients and smokers.

### 3.2. Validation of Common Differentially Methylated Gene Signatures in TCGA Lung Cancer Datasets

DNA methylation profiles from TCGA lung cancer datasets were analyzed to evaluate whether the methylation patterns identified in the literature were also observed in TCGA samples. Methylation patterns in both LUAD and LUSC paired tumor–normal samples were specifically examined. Based on the available data, the following genes were validated: *CDKN2A, CDH13, MGMT, MIR137, DAPK1, RARB, RASSF1A, GSTP1,* and *AHRR*.

**Figure 3:**
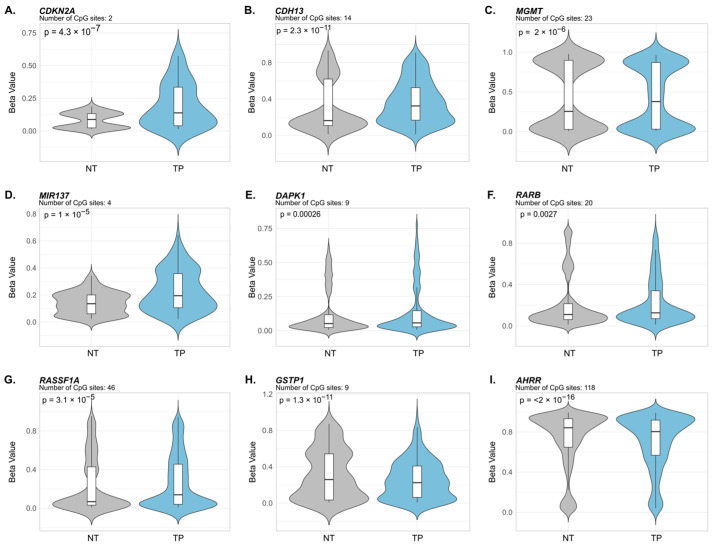
Validation of Common Differentially Methylated Gene Signatures in TCGA Datasets of Lung Adenocarcinoma. Violin plots showing DNA methylation Beta-value distribution of nine commonly differentially methylated genes (panels (**A**–**I**)) in non-tumoral adjacent tissue (NT, gray) versus primary tumor (TP, blue) from the same subjects. Data derived from Illumina HumanMethylation450K arrays of 29 matched tumor–normal pairs from the TCGA-LUAD project. The vertical axis displays Beta-values (0–1), where 1 indicates complete methylation. Box plots within each violin show the median (middle line) and interquartile range for each group. Below each gene name is the number of promoter-associated CpG sites analyzed (except for *AHRR*, where sites are from the gene body) and the FDR-adjusted *p*-value from paired Wilcoxon signed-rank tests assessing statistical significance between matched tumor and normal tissues.

In LUAD, all nine genes showed statistically significant differential methylation. Promoter hypermethylation was demonstrated in *CDKN2A* (*p* = 4.3 × 10^−7^), *CDH13* (*p* = 2.3 × 10^−11^), *MIR137* (*p* = 1 × 10^−5^), *MGMT* (*p* = 2 × 10^−6^), *DAPK1* (*p* = 2.6 × 10^−4^), *RARB* (*p* = 0.0027), and *RASSF1A* (*p* = 3.1 × 10^−5^) (Figure 3A–G). In contrast, *GSTP1* showed statistically significant hypomethylation (*p* = 1.3 × 10^−11^) in tumor tissue compared to adjacent normal tissue (Figure 3H), a finding that contrasts with the hypermethylation previously reported in the literature for this gene in lung cancer. Significant hypomethylation was likewise observed for *AHRR* (*p* < 2 × 10^−16^) (Figure 3I), consistent with the well-established loss of methylation at this locus associated with smoking exposure and lung cancer.

**Figure 4:**
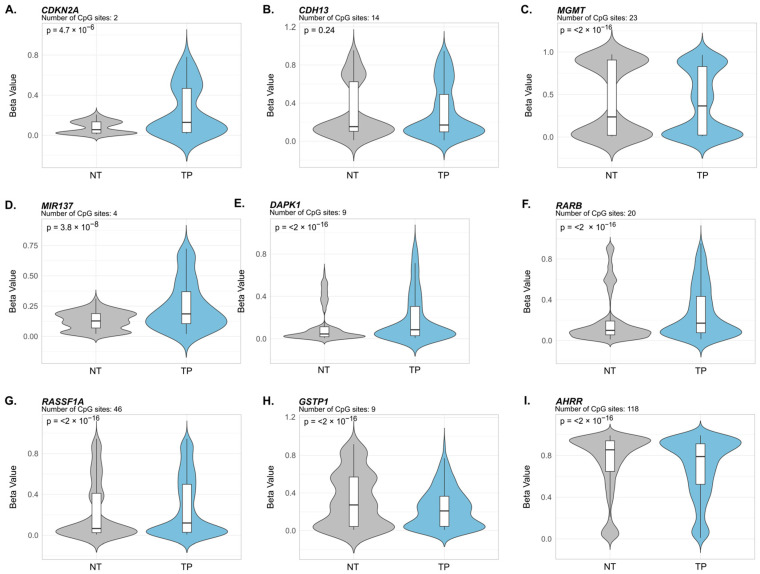
Validation of Common Differentially Methylated Gene Signatures in TCGA Datasets of Lung Squamous Cell Carcinoma. Violin plots showing DNA methylation Beta-value distribution of nine commonly differentially methylated genes (panels (**A**–**I**)) in non-tumoral adjacent tissue (NT, gray) versus primary tumor (TP, blue) from the same subjects. Data derived from Illumina HumanMethylation450K arrays of 40 matched tumor–normal pairs from the TCGA-LUSC project. The vertical axis displays Beta-values (0–1), where 1 indicates complete methylation. Box plots within each violin show the median (middle line) and interquartile range for each group. Below each gene name is the number of promoter-associated CpG sites analyzed (except for *AHRR*, where sites are from the gene body) and the FDR-adjusted *p*-value from paired Wilcoxon signed-rank tests assessing statistical significance between matched tumor and normal tissues.

The LUSC dataset revealed a methylation profile largely consistent with LUAD, though with some notable differences. Eight of the nine candidate genes displayed statistically significant differential methylation (Figure 4). Promoter hypermethylation was confirmed in *CDKN2A* (*p* = 4.7 × 10^−6^), *MGMT* (*p* < 2 × 10^−16^), *MIR137* (*p* = 3.8 × 10^−8^), *DAPK1* (*p* < 2 × 10^−16^), *RARB* (*p* < 2 × 10^−16^), and *RASSF1A* (*p* < 2 × 10^−16^) (Figure 4A,C–G). As in LUAD, *GSTP1* exhibited significant hypomethylation (*p* < 2 × 10^−16^) in tumor tissue (Figure 4H), further contrasting with its reported hypermethylation in COPD and lung cancer in the literature. *AHRR* also showed highly significant hypomethylation (*p* < 2 × 10^−16^) (Figure 4I), confirming its consistent pattern of methylation loss across both lung cancer subtypes. Unlike in LUAD, *CDH13* did not reach statistical significance in LUSC (*p* = 0.24) (Figure 4B), suggesting a subtype-specific methylation pattern for this gene.

Collectively, these findings confirm that most literature-derived methylation signatures are reproducible in TCGA datasets across both lung cancer subtypes. The consistent hypomethylation of *GSTP1* in tumor tissue in both LUAD and LUSC, however, contrasts with its characterization as hypermethylated in lung cancer and COPD in the reviewed literature. This discordance may reflect the scope of the present analysis, which was restricted to promoter-associated CpG sites; methylation changes at non-promoter regions not captured here could account for the differences observed relative to previously reported results.

To further explore the relationship between these epigenetic alterations and tobacco exposure, given that eight of the common genes are shared between tobacco exposure and LuCa conditions (Figure 2), the methylation status of these genes according to smoking category in the TCGA lung cancer dataset was examined as an exploratory analysis. In LUAD tumors, a statistically significant association with smoking categories was observed only for *MIR137* (*p* = 5.39 × 10^−3^), with differences observed primarily between reformed smokers for >15 years (category 2) and current smokers (category 4) (Figure S1A). In LUSC tumors, statistically significant differences across smoking categories were observed for three genes: *RASSF1A* (*p* = 4.46 × 10^−6^), *CDKN2A* (*p* = 4.41 × 10^−2^), and *MIR137* (*p* = 4.61 × 10^−2^), with higher methylation levels observed in reformed and current smokers (categories 2–4) compared to non-smokers (category 1) (Figure S1B–D). The small number of samples per smoking category limits the robustness of these associations, and replication in larger datasets is warranted. Nonetheless, the results provide preliminary evidence of smoking-related methylation differences in a subset of the identified genes, particularly *MIR137*, which showed associations across both LUAD and LUSC subtypes.

### 3.3. Functional Enrichment Analysis of Genes with Changes in DNA Methylation in LuCa, COPD and Cigarette Smoke Exposure

To explore the biological processes and pathways potentially associated with DNA methylation changes in LuCa and its risk factors, GO and KEGG pathway enrichment analyses were performed on 205 genes with methylation changes in lung cancer, 64 in smokers, and 66 in COPD. The results were categorized into BP, MF and KEGG signaling pathways, selecting the top 10 enriched terms with statistically significant *p-*value (*p* < 0.05).

In LuCa, enriched BPs were mainly related to transcription regulation, apoptosis, cell proliferation, cell growth, and epithelial–mesenchymal transition (EMT) (*p* < 0.05). MFs highlighted DNA-binding proteins and developmental regulators. Signaling pathway analysis revealed eight statistically significant pathways, many of which were related to cancer-associated pathways (Figure S2).

In smokers, enrichment BPs included transcriptional regulation, signal transduction, cell differentiation, and apoptosis. Negative regulation of cell proliferation was the only biological process with statistically significant differences. The MFs mirrored those found in lung cancer, while significant signaling pathways were also associated with cancer-related pathways (Figure S3).

In COPD models, although no statistically significant differences were identified, the most enriched BPs, MFs, and KEGG pathways were listed. BPs included transcription regulation and apoptosis, like previous models, and specific processes such as phosphorylation and cellular response to hypoxia. MFs highlighted transferase activity, and signaling pathways shared those related to cancer, in addition to specific pathways such as chemical carcinogenesis, reactive oxygen species, chemokine signaling, MAPK and PI3K-Akt pathways (Figure S4).

Common cellular processes, molecular functions, and signaling pathways associated with differentially methylated genes were subsequently identified across all three studied conditions. The identification of shared enriched terms may reflect common biological contexts across smoking exposure, COPD, and lung cancer.

**Figure 5:**
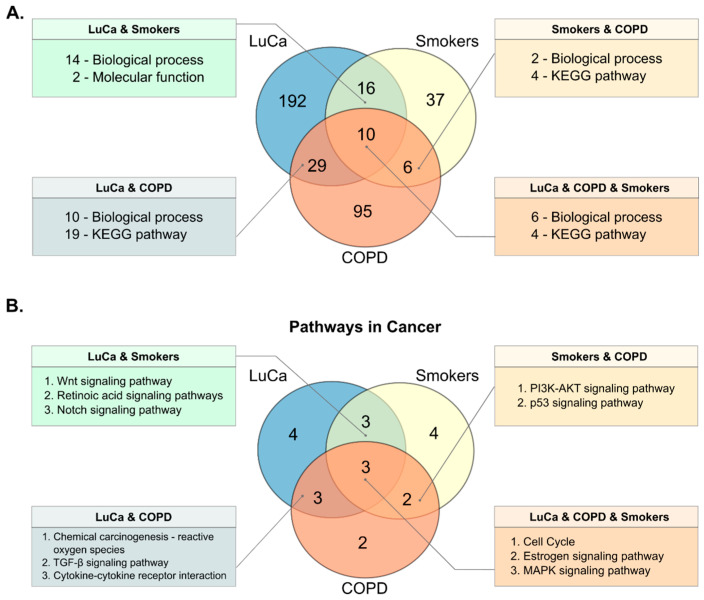
Common biological processes and signaling pathways affected by DNA methylation changes in LuCa, COPD and smoking. (**A**) Venn diagram illustrating the intersection of functional annotation terms. The 10 common terms between LuCa, Smokers and COPD are highlighted, of which six are common biological processes and four are common pathways “Pathways in cancer.” (**B**) Specific cancer-associated pathways altered in each condition, showing shared disruptions in the cell cycle, estrogen signaling, and MAPK signaling pathways. Abbreviations: LuCa, lung cancer; COPD, chronic obstructive pulmonary disease.

Comparative analysis of functional annotations from LuCa (248 annotation terms), tobacco exposure (71 annotation terms), and COPD (143 annotation terms) revealed 10 common mechanisms, comprising six BPs and four signaling pathways (Figure 5A). The shared BPs included signal transduction, transcriptional regulation, and cancer-related hallmarks, such as angiogenesis and apoptotic regulation. The signaling pathways identified among the shared terms were mainly associated with cancer-related pathways. Table S3 details the differentially methylated genes and their corresponding functional annotations shared among LuCa, smokers, and COPD.

Subsequently, we focused on functional annotation analyses. According to the KEGG database, this category includes approximately 21 distinct cancer-associated signaling pathways. Using KEGG, we identified specific cancer-associated pathways linked to differentially methylated genes in each condition. (Table S4). We identified 13 signaling pathways in cancer for the LuCa study models, 12 for smoking, and 10 for COPD. Comparative analysis of these pathways revealed three common pathways across the groups of interest: cell cycle, estrogen signaling, and MAPK pathways (Figure 5B). The differentially methylated genes involved in these pathways are detailed in Table 2.

### 3.4. Transcription Factor Binding Site Prediction in Differentially Methylated Genes

A transcription factor binding motif overrepresentation analysis on the genes with DNA methylation changes was performed using the g: GOSt function within the g: Profiler tool. The analysis was focused on 135 genes involved in shared biological processes and signaling pathways across the three study groups (Table S3).

**Figure 6:**
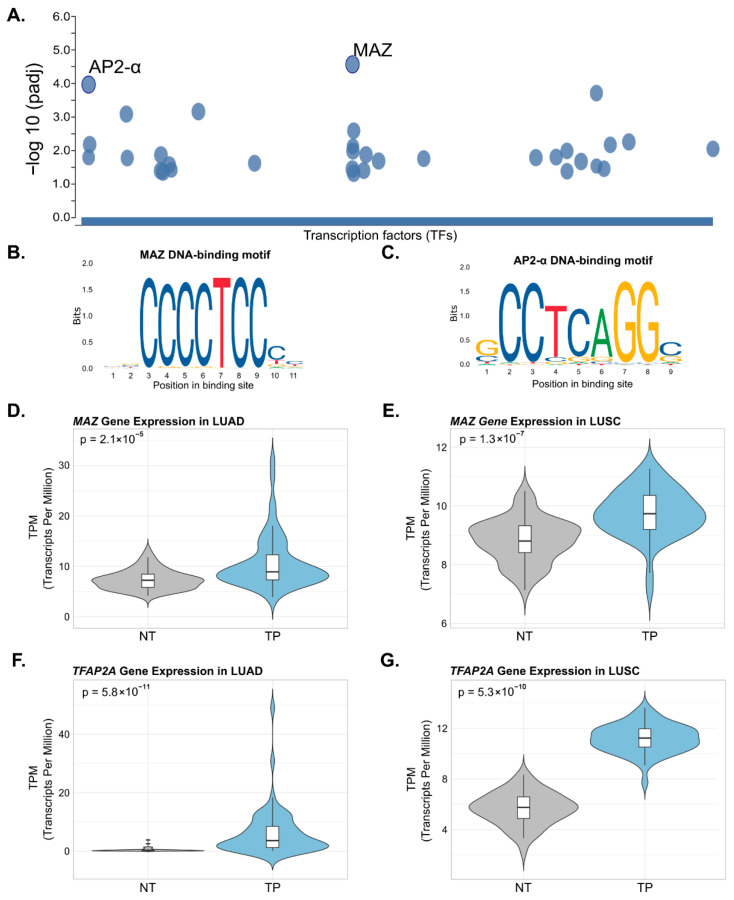
Transcription factor motif enrichment and expression analysis in lung cancer. (**A**) Functional annotation analysis identifying transcription factor regulatory motifs in methylated genes associated with common processes and pathways across LuCa, smokers and COPD. (**B**,**C**) Identification of the MAZ and AP2-α transcription factor binding motifs, derived from the JASPAR database. (**D**,**E**) Violin plots showing *MAZ* mRNA expression (TPM) in non-tumoral adjacent tissue (NT, gray) versus primary tumor (TP, blue) in matched LUAD and LUSC samples. (**F**,**G**) Violin plots showing *TFAP2A* mRNA expression (TPM) in NT versus TP in matched LUAD and LUSC samples. Data derived from RNA-seq STAR-Counts workflow from TCGA-LUAD and TCGA-LUSC projects. Box plots within each violin show the median (middle line) and interquartile range. *p*-values were obtained from paired Wilcoxon signed-rank tests.

The regulatory motif analysis identified 28 transcription factors (Table S5), among which only MAZ (*MYC Associated Zinc Finger Protein*) and AP-2α showed statistically significant motif enrichment (Figure 6A). Binding motif characterization through the JASPAR database (Figure 6B,C) revealed that the MAZ recognition sequence was present in 72.5% of the analyzed genes, while AP-2α binding sites were identified in 70% of the evaluated genes. These findings indicate a potential enrichment of binding motifs for these transcription factors among the differentially methylated genes analyzed.

To assess whether these transcription factors are also transcriptionally altered in lung cancer, their mRNA expression was evaluated in paired tumor–normal samples from TCGA-LUAD and TCGA-LUSC cohorts. *MAZ* expression was significantly upregulated in tumor tissue relative to adjacent normal tissue in both LUAD (*p* = 2.1 × 10^−5^, Figure 6D) and LUSC (*p* = 1.3 × 10^−7^, Figure 6E). Similarly, *TFAP2A* showed significant upregulation in tumor tissue in both subtypes, with a particularly pronounced increase in LUAD (*p* = 5.8 × 10^−11^, Figure 6F) and LUSC (*p* = 5.3 × 10^−10^, Figure 6G). These findings suggest that both transcription factors are not only enriched as potential regulators of differentially methylated regions but are also transcriptionally active in lung tumor tissue, supporting their potential functional relevance in lung carcinogenesis.

## 4. Discussion

Epigenetic regulation, encompassing DNA methylation and non-coding RNAs such as microRNAs, long non-coding RNAs, and piRNAs, constitutes a central layer controlling gene expression and modulating responses to environmental exposures in lung disease [72,73,74]. These regulatory mechanisms interact dynamically with the chromatin remodeling process, collectively shaping the complex epigenetic landscape that may contribute to lung tumor development and therapeutic response [72,73]. Tobacco smoke, a potent environmental modifier, significantly alters DNA methylation patterns, contributing to chronic pulmonary disorders, including lung cancer and COPD [75]. Such epigenetic alterations have been proposed as potential biomarkers for early diagnosis, prognosis and therapy monitoring [76]. In COPD, DNA methylation further modulates genes involved in oxidative stress responses and chronic inflammation, underscoring its role in disease progression [56].

In this study, based on the previously reported literature, differentially methylated genes and shared biological processes were identified between chronic lung pathologies and environmental exposure to cigarette smoke (Figure 1 and Figure 2). These differential methylation signatures were further examined using computational analyses of the TCGA LUAD and LUSC datasets (Figure 3 and Figure 4). It should be noted that the TCGA validation focused exclusively on lung cancer datasets, as comparable genome-wide methylation datasets for COPD are not available. Our findings are consistent with the possibility that aberrant DNA methylation in the respiratory epithelium may be associated with processes linked to malignant transformation [77]. These observations provide a framework for understanding the increased risk of lung cancer in COPD patients and chronic smokers, although additional experimental studies are required to establish causal relationships.

In this context, the findings highlight that tobacco exposure induces epigenetic alterations that show notable similarity to those described in LuCa, particularly regarding the hypermethylation of tumor suppressor genes such as *CDKN2A, CDH13, MGMT, MIR137, DAPK1, RARB,* and *RASSF1A*, linking smoking-related molecular changes to lung carcinogenesis (Figure 2).

Of particular interest is the hypomethylation of *LINE-1* elements in both lung cancer and smokers (Figure 2B). The hypomethylation of these retrotransposons is a characteristic cancer marker associated with genomic instability [78,79]. Their detection in the peripheral blood of cigarette users, including both conventional [17] and electronic cigarettes [80], supports the relevance of these epigenetic alterations in smoking-associated molecular changes.

Finally, the hypomethylation of the *AHRR* gene was identified as the only differentially methylated gene common across all three study models (Figure 2B). *AHRR* is a critical regulator of the aryl hydrocarbon receptor (AhR) signaling pathway, and its loss of methylation has been correlated with lung function parameters and nicotine dependence. This epigenetic signature has been widely reported as a marker of tobacco exposure, suggesting a potential molecular connection between smoking exposure and lung carcinogenesis [81,82,83]. It should be noted, however, that *AHRR* methylation patterns may vary depending on the specific CpG sites and cell type analyzed. In this regard, Monick et al. [49] reported bidirectional methylation changes at distinct regions of the *AHRR* gene body in a cell type-dependent manner: hypomethylation was observed at cg05575921, cg14817490, cg14454127, and cg03991871 in lymphoblast, whereas hypermethylation was detected at cg25648203 and cg04135110 exclusively in alveolar macrophage. Importantly, the differential methylation at cg05575921 was functionally associated with *AHRR* gene expression, suggesting this CpG site play a functional role in transcriptional regulation. Therefore, hypomethylation—particularly at cg05575921—represents the most consistently validated signal associated with smoking exposure and lung disease, and is therefore emphasized in the present analysis.

Tobacco smoke alters DNA methylation patterns through several molecular mechanisms, as outlined by Lee et al. [75]. First, carcinogens in smoke induce DNA double-strand breaks, activating DNA repair pathways that recruit DNA methyltransferase 1 (DNMT1), leading to methylation of CpG regions near repair sites [84,85,86]. Second, nicotine activates nicotinic acetylcholine receptors, triggering downstream signaling that activates cAMP response element binding protein (CREB), a transcription factor that regulates DNMT1 expression [87,88]. Additionally, tobacco smoke enhances the activity of transcription factors like Sp1, which suppresses de novo methylation in GC-rich promoter regions [89,90]. Finally, carbon monoxide-induced tissue hypoxia upregulates Hypoxia-Inducible Factor 1-alpha (HIF-1α), increasing the expression of MAT2A, an enzyme that synthesizes the methyl group donor S-adenosylmethionine [91].

Recent studies have further expanded the understanding of epigenetic regulation in smoking-related pulmonary diseases, highlighting multiple layers of gene expression control. Epigenetic mechanisms encompass DNA methylation, non-coding RNAs, and histone-modifying enzymes such as SIRT6. Exosomal lncRNA MEG3, for example, promotes M1 macrophage polarization and pyroptosis via m6A RNA methylation, providing a potential mechanistic link between cigarette smoke exposure and inflammatory responses in COPD. Concurrently, SIRT6-mediated histone deacetylation attenuates NF-κB-driven inflammation in pulmonary endothelial cells, emphasizing the role of chromatin modification in regulating gene expression under stress. Together, these observations underscore that multiple epigenetic layers may act in concert to modulate inflammation, tissue remodeling, and potentially the increased lung cancer risk observed in chronic smokers and COPD patients [92,93].

The differential methylation analysis between LuCa and COPD models identified the hypermethylation of the *GSTP1* gene (Figure 2A). This gene encodes an essential isoform for the detoxification of xenobiotics and cellular protection against oxidative stress. Previous studies have reported GSTP1 hypermethylation in both lung cancer and COPD, suggesting that epigenetic alterations affecting xenobiotic detoxification pathways may be involved in smoking-related pulmonary diseases [45,94]. However, the TCGA analysis performed in the present study did not show significant differential methylation for this gene in lung cancer datasets, indicating that these findings should be interpreted cautiously and may reflect differences between study populations or analytical approaches.

An important observation from this integrative analysis was the apparent functional convergence at the level of cancer signaling pathways across the three analyzed models (Figure 5 and Table S3). This suggests that, although the differentially methylated genes vary across the three conditions, the results point toward convergence on biological pathways associated with tumor development. Among these, the cell cycle, MAPK, and estrogen signaling pathways stand out (Figure 5B), highlighting potential biological connections between these risk factors and lung tumorigenesis.

**Figure 7:**
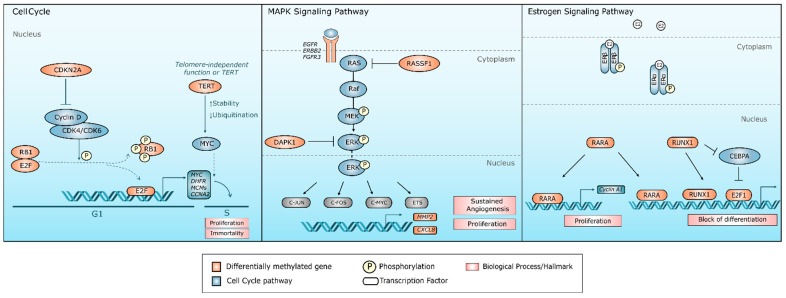
Hypothesized biological model illustrating the impact of DNA methylation alterations in lung cancer (LuCa), chronic obstructive pulmonary disease (COPD) and smoking. The figure summarizes shared molecular pathways displaying differential methylation patterns across LuCa, COPD and smoking conditions, including alterations in cell cycle regulation, MAPK signaling and estrogen signaling pathways. The model was constructed based on information retrieved from the KEGG database.

Based on these enrichment results, we constructed a conceptual model summarizing potential signaling pathways that may be influenced by the differentially methylated genes identified in this study (Figure 7). This model should be interpreted as a hypothesis-generating framework illustrating possible biological connections between smoking exposure, COPD, and lung cancer, rather than as a mechanistic demonstration.

Upon further exploration of these pathways, the analysis of cell cycle signaling (Figure 7) revealed the differential methylation of key genes such as *CDKN2A*, *RB1*, *E2F1*, and *TERT* (Table 2). Under normal conditions, *CDKN2A* antagonizes the formation of the cyclin D/CDK4-6 complex. However, its hypermethylation reduces its expression, allowing the cyclin D/CDK4 complex to phosphorylate the retinoblastoma protein (pRB1) [95]. This phosphorylation releases the transcription factor E2F, which activates the transcription of genes essential for cell cycle progression. Similarly, *TERT* potentiates oncogenesis by enhancing the stability and function of *MYC*, thereby increasing the activity of target genes involved in cell cycle regulation and proliferation [96].

Additionally, it was found that the MAPK signaling pathway (Figure 7), which includes the RAS-RAF-MEK-ERK cascade, controls key processes in cancer such as proliferation, cell survival, and angiogenesis [97]. Notably, the hypermethylation of negative regulators of this signaling pathway, such as *RASSF1A* and *DAPK1*, was identified [98]. In the lung cancer model, differential methylation of tyrosine kinase receptors (*EGFR*, *ERBB2*, and *FGFR3*), which are necessary for Ras activation, was observed.

Finally, regarding the estrogen signaling pathway (Figure 7), specific patterns of differential methylation were identified. Among these, the hypomethylation of *RUNX1*, a modulator that directly interacts with the estrogen receptor (ESR1) to regulate estrogen-sensitive genes and the expression of *E2F1*, a critical factor for cell proliferation, stands out [99]. In parallel, the hypomethylation of *RARA* was observed, which, upon binding to retinoic acid, forms complexes with estrogen receptors ERα and ERβ at estrogen response elements, thereby regulating cell proliferation genes such as *cyclin A1* [100].

A significant finding was the identification of MAZ and AP-2α binding domains in the regulatory regions of genes that showed DNA methylation changes associated with LuCa, COPD, and smoking exposure (Figure 6). Previous studies have demonstrated that MAZ promotes tumor progression and metastasis through two mechanisms: direct transcriptional regulation by binding to promoter regions and synergistic interactions with other transcription factors. Its characteristic interaction with GC-rich sites aligns with the identification of MAZ as a regulator of differentially methylated regions, supporting the biological relevance of these findings. As a dual regulator, MAZ can act as either an activator or inhibitor of the transcription of critical genes, including proto-oncogenes such as c-MYC, Ras genes, and genes essential for tumor progression, such as VEGF and hTERT [101].

Similarly, AP-2α, a member of the TFAP2 family, contains a conserved DNA-binding domain that recognizes GCC(N3)GGC sequences in promoter and enhancer regions, a specificity that supports its identification as a potential regulator of differentially methylated regions [102]. This transcription factor influences epigenetic regulation by modifying chromatin accessibility through interactions with the NuRD complex and EZH2. Its role in tumorigenesis is extensive, encompassing the regulation of EMT, interactions with the tumor microenvironment, cell cycle control, DNA damage repair, and various signaling pathways, including those related to ER- and ERBB2, as well as ferroptosis [103].

For instance, activator protein (AP) transcription factors, including TFAP2A (AP 2α) and AP 1 family members, have been shown to regulate cytokine expression from noncanonical promoters in chronic airway disease, with increased AP 2 and AP 1 expression observed in COPD lung tissue [104]. Genome-wide DNA methylation profiling in COPD bronchoalveolar lavage (BAL) cells has revealed widespread differentially methylated positions with functional enrichment for transcription factor activity and regulatory pathways, suggesting that epigenetic alterations may influence gene regulation by modulating transcription factor binding at regulatory regions [105]. Consistently, integrative epigenomic analyses of lung fibroblasts from COPD patients have identified enrichment of methylation-sensitive transcription factor motifs within differentially methylated regions, supporting a role for DNA methylation in shaping transcriptional regulatory networks in COPD [106]. Moreover, aberrant DNA methylation at promoters of differentiation-associated transcription factors such as SPDEF and FOXA2 has been linked to airway epithelial remodeling and mucus hypersecretion in COPD patients [107]. These observations support the hypothesis that enrichment of transcription factor binding motifs in differentially methylated regions may reflect regulatory mechanisms integrating environmental exposures, such as tobacco smoke or air pollution, with epigenetic alterations relevant to chronic pulmonary disease and lung cancer risk.

The identification of MAZ and AP-2α binding motifs suggests that these transcription factors may contribute to the regulation of differentially methylated regions. Furthermore, this regulatory mechanism appears to be associated with both environmental risk factors (such as tobacco smoke exposure) and processes related to lung tumorigenesis. Further experimental studies will be required to clarify the biological relevance of these transcription factors in the context of lung cancer and smoking-associated epigenetic alterations.

## 5. Limitations

This study presents several limitations that should be considered when interpreting the results. First, the identification of differentially methylated genes was based on literature mining across multiple independent studies. Consequently, the compiled dataset may include heterogeneity related to differences in experimental platforms, sample types, study designs, and analytical thresholds used across the original publications. Therefore, the present work should be interpreted as an integrative and descriptive mapping of methylation alterations reported in the literature rather than a formal quantitative meta-analysis.

Second, the selection of genes relied on statistically significant findings reported in individual studies (*p* < 0.05), without applying a unified multiple-testing correction across the aggregated dataset. Although this approach enabled the integration of findings from a broad body of literature, it may have influenced the number of genes identified as differentially methylated.

A key limitation of this study lies in the exclusion of epigenome-wide association studies (EWAS) that lacked biological validation. While EWAS constitute a powerful and comprehensive approach for identifying differentially methylated regions, their reliance on high-dimensional statistical associations may yield findings with limited functional interpretability. By restricting our analysis to studies incorporating biological validation—such as concordant gene expression changes or functional assays—we may have omitted potentially relevant methylation signals identified in large, well-powered datasets. However, this conservative approach was intentionally adopted to prioritize epigenetic alterations with greater biological plausibility and mechanistic relevance, thereby reducing the risk of false-positive findings driven by confounding factors, cell-type heterogeneity, or multiple testing burden. As a result, although this criterion may limit the breadth of included evidence, it strengthens the overall robustness, interpretability, and translational potential of the identified shared methylation signatures.

Additionally, the validation analysis using TCGA datasets was based on a relatively limited number of paired samples (29 LUAD and 40 LUSC), which may reduce the statistical power to detect subtle methylation differences. Nevertheless, the use of paired tumor–normal samples allowed controlled comparisons within the same individuals, providing additional support for several of the methylation patterns identified. Furthermore, although TCGA data enabled validation of methylation patterns associated with lung cancer, this resource does not include specific datasets for COPD, which prevented the direct validation of COPD-related methylation alterations.

Finally, the present study is primarily based on integrative computational analyses. Therefore, further experimental studies will be necessary to explore the biological and mechanistic relevance of the identified epigenetic signatures.

Despite these limitations, the integrative approach employed in this work allowed the identification of shared epigenetic patterns and convergent biological processes associated with smoking exposure, COPD, and lung cancer. These findings provide a useful framework for future studies aimed at validating potential epigenetic biomarkers and further exploring the molecular links between these pulmonary conditions.

## 6. Conclusions

Our integrative analysis indicates the presence of shared epigenetic alterations associated with tobacco exposure, chronic obstructive pulmonary disease (COPD), and lung cancer. These alterations include recurrent hypermethylation of tumor suppressor genes such as *CDKN2A, CDH13, MGMT, MIR137, DAPK1, RARB*, and *RASSF1A,* together with hypomethylation of repetitive elements including *LINE-1*, reflecting patterns of epigenomic dysregulation associated with smoking-related pulmonary disease. Notably, altered methylation of *AHRR*, a well-established marker of tobacco exposure, may represent a potential epigenetic indicator associated with cigarette smoke exposure and pulmonary disease processes.

Genes exhibiting altered methylation patterns were enriched in pathways central to tumorigenesis, including cell cycle regulation, MAPK signaling, and estrogen signaling, suggesting that tobacco-associated epigenetic alterations may be related to molecular programs relevant to both chronic lung disease and cancer development. Furthermore, differentially methylated regions shared across the evaluated conditions showed enrichment of binding motifs for transcription factors such as MAZ and AP-2α, which preferentially bind GC-rich regulatory regions, suggesting a possible relationship between transcriptional regulatory motifs and DNA methylation patterns observed in smoking-related pulmonary disease.

Collectively, these findings support the concept that epigenetic alterations may represent a common molecular layer associated with tobacco exposure with chronic pulmonary disease and lung carcinogenesis. By identifying overlapping methylation changes across these conditions, this work contributes to a broader understanding of the molecular connections between smoking, COPD, and lung cancer, and provides a framework for future studies exploring epigenetic biomarkers for risk assessment and early detection.

## Figures and Tables

**Table 1:** Genes with common aberrant methylation patterns in lung cancer, smokers, and COPD.

		Sample Type		
Gene	Methylation Status	Lung Cancer	Smokers	COPD
*AHRR*	Hypomethylated	Whole blood [48]	BAL, whole blood, and saliva [49,50,51].	Primary airway epithelial cells [52].
*L1TD1*		Tumor tissue [53].	Respiratory epithelia cell lines [54].	
*GSTP1*	Hypermethylated	Tumor tissue and serum [55].	-	Bronchial brushing cells [56].
*CDH13*		Tumor tissue and plasma [57].	Respiratory epithelial cell line [58].	-
*CDKN2A*		Tumor tissue, plasma, BAL, EBC. Lung cancer cell lines [59,60,61,62,63].	Bronchial brushing cells and sputum [64].	-
*MGMT*		Tumor tissue, plasma, and sputum [65,66].	Bronchial brushing cells and sputum [64].	-
*MIR137*		Tumor tissue and lung cancer cell lines [67].	Oral mucosa cells [68].	-
*DAPK1*		Tumor tissue and serum [55].	Bronchial brushing cells and EBC [64,69].	-
*RARB*		Tumor tissue [62].	Respiratory epithelial cell line [54].	-
*RASSF1A*		Tumor tissue, plasma, BAL, and sputum [66,70,71].	Bronchial brushing cells, EBC, sputum, and respiratory epithelial cell line [64,69].	-

**Table 2:** Common cancer signaling pathways for lung cancer, conventional cigarette exposure and COPD.

Term	Model	Differentially Methylated Genes
Cell Cycle	LuCa	*RB1, CDKN2A, TERT*
	Smokers	*CDKN2A*
	COPD	*E2F1*
Estrogen signaling pathway	LuCa	*RUNX1*
	Smokers	*RARA*
	COPD	*E2F1*
MAPK signaling pathway	LuCa	*EGFR, ERBB2, FGFR3, RASSF1A, DAPK1, CXCL8*
	Smokers	*DAPK1*
	COPD	*MMP2*

## Data Availability

The data that support the findings of this study are available from the corresponding author upon reasonable request.

## Supplementary Materials

The following supporting information can be downloaded at: https://www.mdpi.com/article/10.3390/cancers18081273/s1. Figure S1. Beta values of methylation sites across different smoking status groups in lung cancer subtypes. (**A**) Violin plots showing methylation levels (beta values) of MIR137 gene in lung adenocarcinoma (LUAD) samples. (**B**) Methylation patterns of RASSF1A gene in lung squamous cell carcinoma (LUSC) samples. (**C**) Methylation patterns of CDKN2A gene in LUSC samples. (**D**) Methylation patterns of MIR137 gene in LUSC samples. Samples were categorized according to TCGA clinical annotation into four smoking groups: non-smokers (category 1), reformed smokers for >15 years (category 2), reformed smokers for ≤15 years (category 3), and current smokers (category 4). Horizontal bars indicate statistical significance between groups (* *p* < 0.05, ** *p* < 0.01, *** *p* < 0.001). Abbreviations: LUAD, lung adenocarcinoma; LUSC, lung squamous cell carcinoma; KW, Kruskal-Wallis test. Figure S2. Functional annotation of genes exhibiting altered methylation patterns in LuCa. Results from functional enrichment analysis of differentially methylated genes in lung cancer (LuCa) revealing significantly overrepresented Gene Ontology (GO) biological processes, molecular functions, and Kyoto Encyclopedia of Genes and Genomes (KEGG) pathways. Analysis was performed using the Database for Annotation, Visualization and Integrated Discovery (DAVID) tool. Statistical significance is indicated as follows: * *p* < 0.05, ** *p* < 0.01, *** *p* < 0.001. Figure S3. Functional annotation of genes with altered methylation patterns associated with smoking exposure. Results from functional enrichment analysis of smoking-related differentially methylated genes revealing significantly overrepresented Gene Ontology (GO) biological processes, molecular functions, and Kyoto Encyclopedia of Genes and Genomes (KEGG) pathways. Analysis was performed using the Database for Annotation, Visualization and Integrated Discovery (DAVID) tool. Statistical significance is indicated as * *p* < 0.05. Figure S4. Functional annotation of genes with altered methylation patterns in COPD. Results from functional enrichment analysis of differentially methylated genes in chronic obstructive pulmonary disease (COPD), highlighting Gene Ontology (GO) biological processes, molecular functions, and Kyoto Encyclopedia of Genes and Genomes (KEGG) pathways. Analysis was performed using the Database for Annotation, Visualization and Integrated Discovery (DAVID) tool. Table S1. Selected Studies Analyzing DNA Methylation in Lung Cancer, COPD, and Smoking. Table S2. List of Genes with Statistically Significant DNA Methylation Changes across Study Groups. Table S3. Common biological processes and signaling pathways associated with differentially methylated Genes in Lung Cancer, COPD, and Smoking. Table S4. Pathways in Cancer Associated with Differentially Methylated Genes Identified Using KEGG. Table S5. Identify transcription factor binding sites (TFBS) in differential methylation genes.

## Abbreviations

LuCa	lung cancer
COPD	chronic obstructive pulmonary disease
LUAD	lung adenocarcinoma
LUSC	lung squamous cell carcinoma
5mC	5-methylcytosine
DNMT	DNA methyltransferase
TSS	transcription start site
5′UTR	5′ untranslated region
BP	biological processes
MF	molecular functions
CDKN2A	cyclin dependent kinase inhibitor 2A
CDH13	cadherin 13
MGMT	O-6-methylguanine-DNA methyltransferase
MIR137	microRNA 137
DAPK1	death associated protein kinase 1
RARB	retinoic acid receptor beta
RASSF1A	Ras association domain family member 1A
GSTP1	glutathione S-transferase pi 1
AHRR	aryl hydrocarbon receptor repressor
EMT	epithelial–mesenchymal transition
MAZ	MYC associated zinc finger protein
