# Supplementary material for: Molecular Links Between Smoking, COPD, and Lung Cancer: A DNA Methylation Perspective
Source: Cancers (Basel). 2026 Apr 17;18(8):1273. doi: 10.3390/cancers18081273 (PMC13115189; doi:10.3390/cancers18081273)
Supplement: Supplementary file 1 [file cancers-18-01273-s001.zip › cancers-4193889-supplementary.docx]

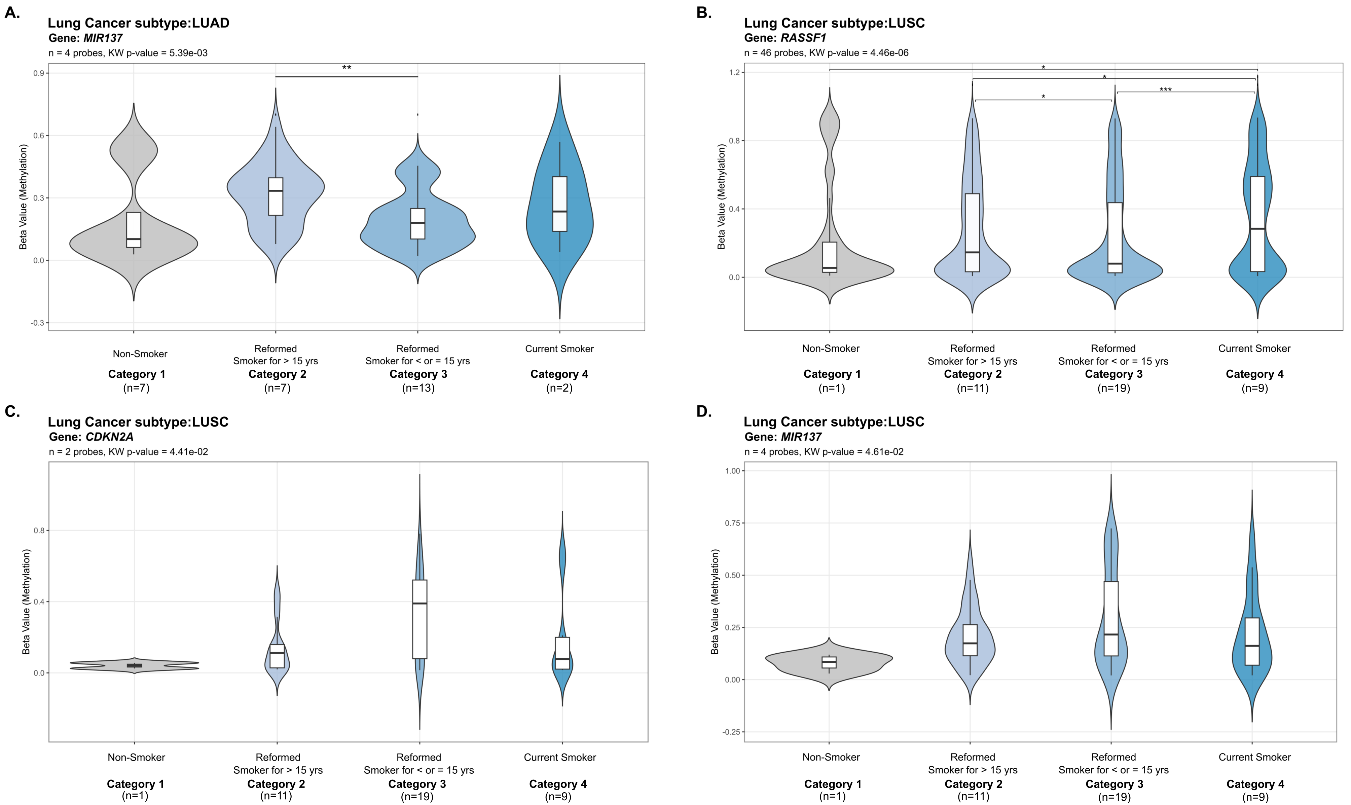


**Supplementary Figure S1. Beta values of methylation sites across different smoking status groups in lung cancer subtypes.** (A) Violin plots showing methylation levels (beta values) of *MIR137* gene in lung adenocarcinoma (LUAD) samples. (B) Methylation patterns of *RASSF1A* gene in lung squamous cell carcinoma (LUSC) samples. (C) Methylation patterns of *CDKN2A* gene in LUSC samples. (D) Methylation patterns of *MIR137* gene in LUSC samples. Samples were categorized according to TCGA clinical annotation into four smoking groups: non-smokers (category 1), reformed smokers for >15 years (category 2), reformed smokers for ≤15 years (category 3), and current smokers (category 4). Horizontal bars indicate statistical significance between groups (*p < 0.05, **p < 0.01, ***p < 0.001). Abbreviations: LUAD, lung adenocarcinoma; LUSC, lung squamous cell carcinoma; KW, Kruskal-Wallis test.


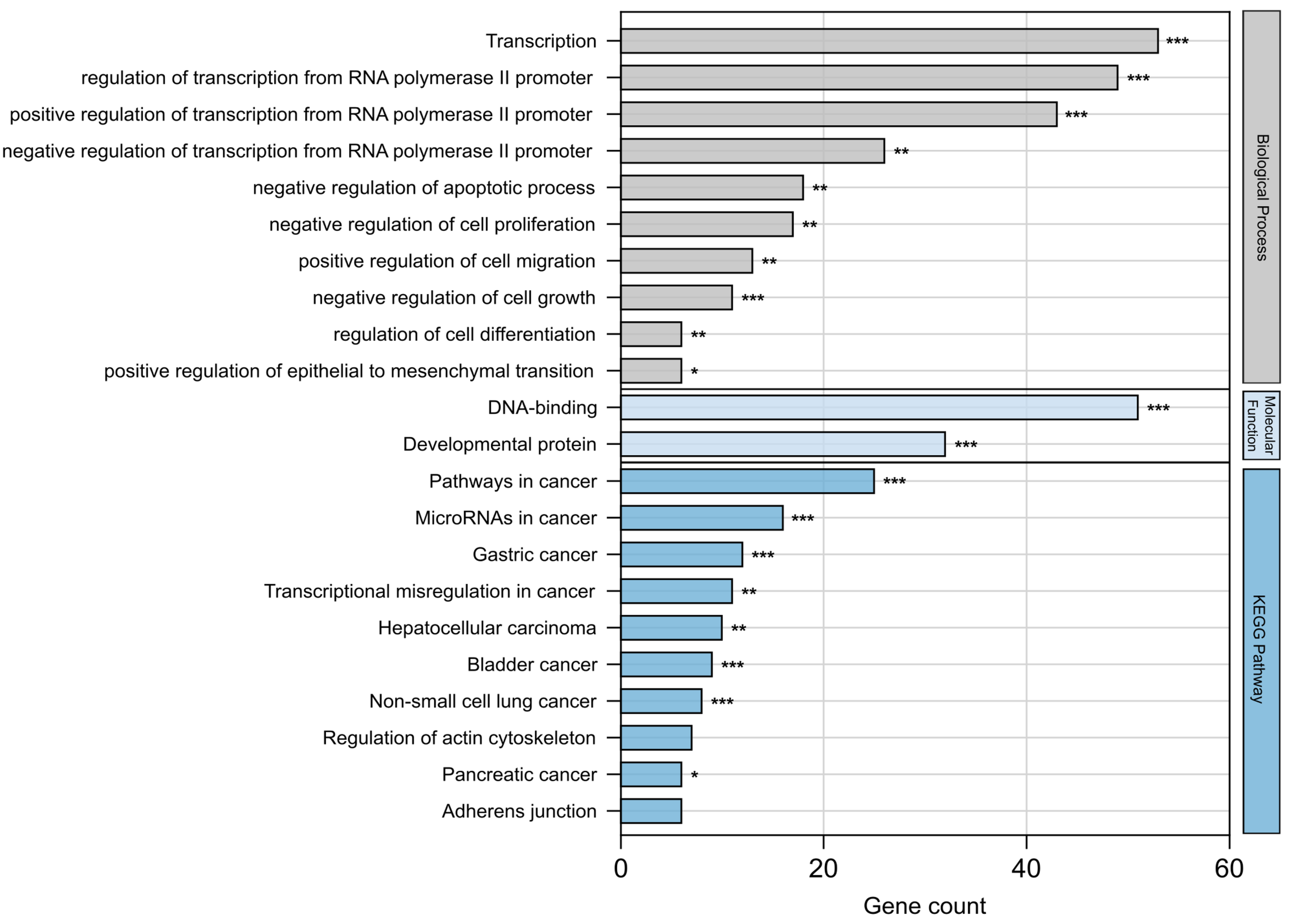


**Supplementary Figure S2. Functional annotation of genes exhibiting altered methylation patterns in LuCa.** Results from functional enrichment analysis of differentially methylated genes in lung cancer (LuCa) revealing significantly overrepresented Gene Ontology (GO) biological processes, molecular functions, and Kyoto Encyclopedia of Genes and Genomes (KEGG) pathways. Analysis was performed using the Database for Annotation, Visualization and Integrated Discovery (DAVID) tool. Statistical significance is indicated as follows: *p < 0.05, **p < 0.01, ***p < 0.001.


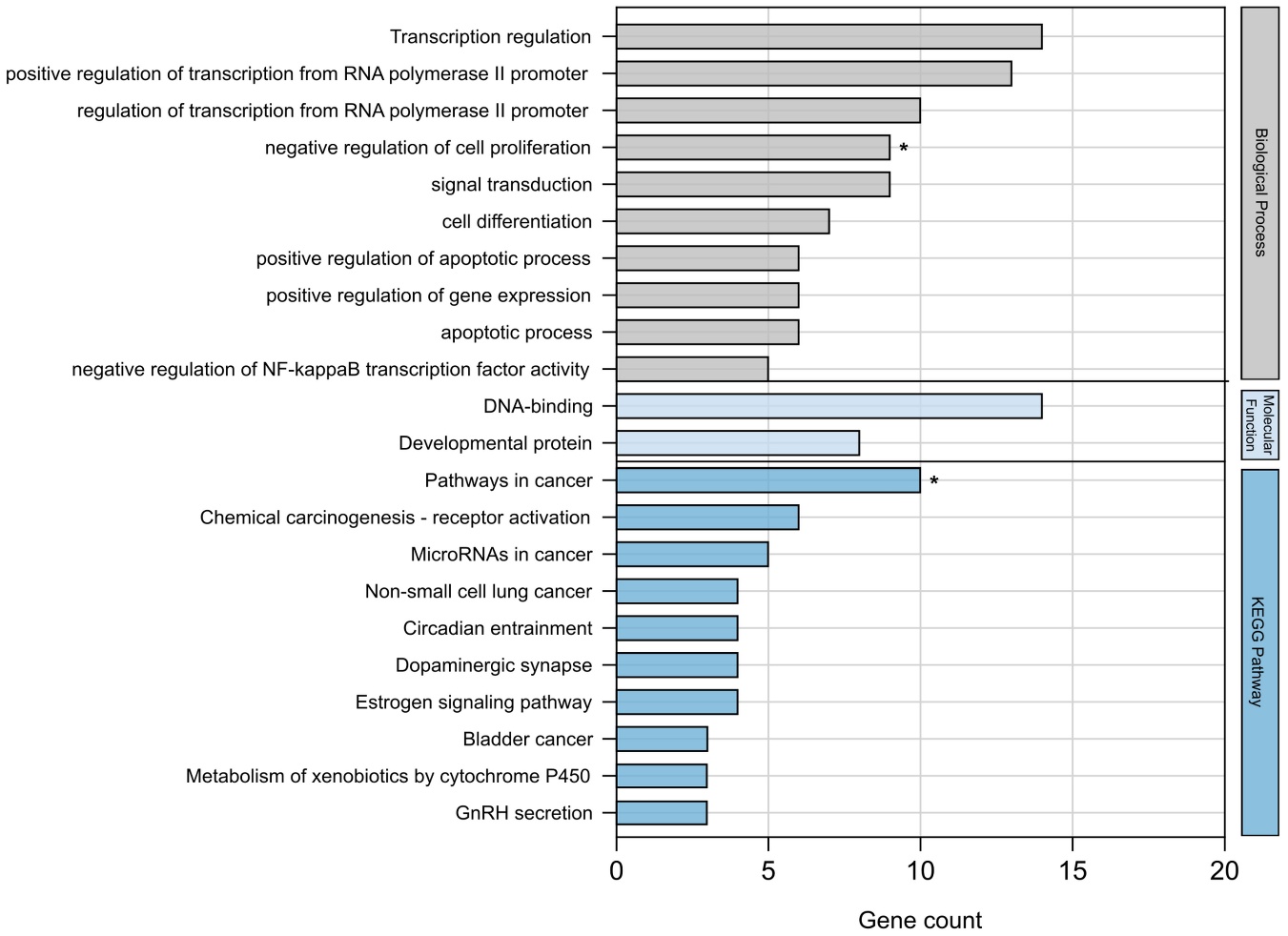


**Supplementary Figure S3. Functional annotation of genes with altered methylation patterns associated with smoking exposure.** Results from functional enrichment analysis of smoking-related differentially methylated genes revealing significantly overrepresented Gene Ontology (GO) biological processes, molecular functions, and Kyoto Encyclopedia of Genes and Genomes (KEGG) pathways. Analysis was performed using the Database for Annotation, Visualization and Integrated Discovery (DAVID) tool. Statistical significance is indicated as *p < 0.05.


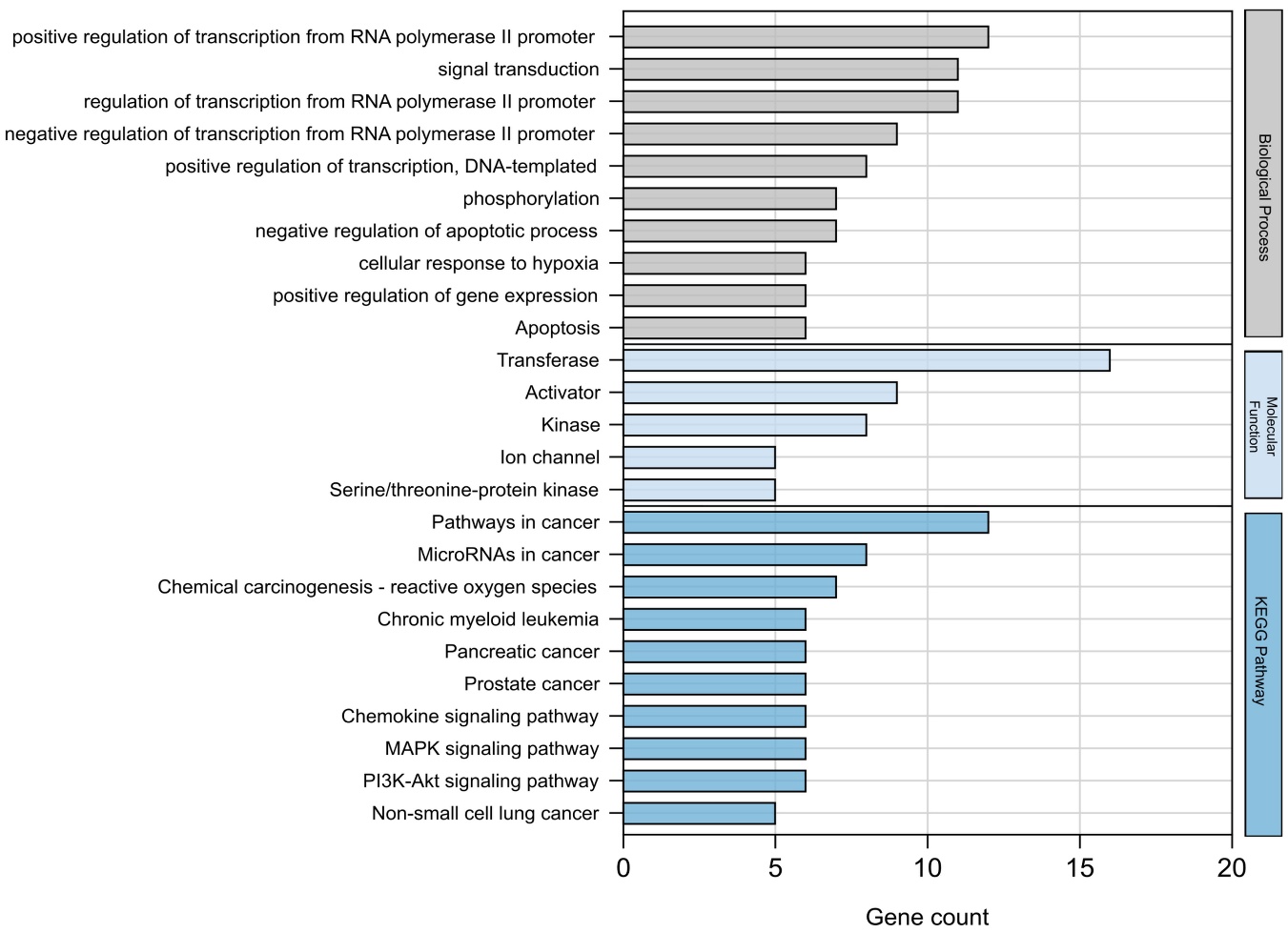


**Supplementary Figure S4. Functional annotation of genes with altered methylation patterns in COPD.** Results from functional enrichment analysis of differentially methylated genes in chronic obstructive pulmonary disease (COPD), highlighting Gene Ontology (GO) biological processes, molecular functions, and Kyoto Encyclopedia of Genes and Genomes (KEGG) pathways. Analysis was performed using the Database for Annotation, Visualization and Integrated Discovery (DAVID) tool.

| **Supplementary Table S1.** Selected Studies Analyzing DNA Methylation in Lung Cancer, COPD, and Smoking. | | | | |
| --- | --- | --- | --- | --- |
| **Model** | **Sample Type** | **Hypermethylated genes** | **Hypomethylated genes** | **PMID** |
| LuCa | Bronchial Washing Fluid | *PCDHGA12, CDO1* |  | 38225788 |
| LuCa | Lung biopsy and cell lines | *OTX2, P16* |  | 37969391 |
| LuCa | Plasma | *SHOX2, PTGER4* |  | 37969955 |
| LuCa | Bronchoalveolar Lavage |  |  | 37682182 |
| LuCa | Lung biopsy and plasma |  | *KIFC1* | 37666088 |
| LuCa | Cell Lines | *PDL-1* |  | 36980795 |
| LuCa | Peripheral blood |  | *FUT7* | 36463240 |
| LuCa | Plasma | *SHOX2, PTGER4* |  | 33662689 |
| LuCa | Tissue and liquid biopsy datasets | *RPTOR, TRPV2, AMPD3, TRAF1, CXXC5, SPON2* | *VGLL4, LYPD8, VPS13D* | 35126793 |
| LuCa | Tumor biopsy and cell lines | *DIO3OS* |  | 34761103 |
| LuCa | Tumor biopsy | *SHOX2* |  | 35117218 |
| LuCa | Tumor biopsy | *DAPK1, P16, RASSF1A* |  | 32914849 |
| LuCa | Plasma and urine | *DO1, TAC1, HOXA7, HOXA9, SOX17, ZFP42* |  | 32430478 |
| LuCa | Tumor tissue and plasma | *HOXA9 , RASSF1A* |  | 31546933 |
| LuCa | Tumor biopsy | *MYF6, SIX6, RXRG, LHX1, RASSF1A, TERT* |  | 31526458 |
| LuCa | Tumor biopsy |  | *MIR130A* | 31524549 |
| LuCa | Cell Lines | *MIR9-1* |  | 31158449 |
| LuCa | Tumor tissue and plasma | *B3GAT2, BCAR1, HLF, HOPX, HOXD11, MIR1203, MYL9, SLC9A3R2, SYT5,VTRNA1-3* |  | 30867848 |
| LuCa | Tumor biopsy and Cell Lines | *SPOP* |  | 30607139 |
| LuCa | Cell Lines | *HOXA1* |  | 30596070 |
| LuCa | Plasma | *WT1,CDKN2A, HOXA9, PITX2, CALCA, RASSF1A, CDH13,DLEC1* |  | 30516882 |
| LuCa | Buffy Coat | *PC, B3GNTL1, F2RL3, DOPEY2, KPNA7, KIAA0087, MYO15A, CA5A, PDZD3, CSHL1, MYO9B, CRTAM, OCA2, FES.* | *AHRR* | 30425263 |
| LuCa | Tumor biopsy | *STXBP6, BCL6B, FZD10, and HSPB6* |  | 30286088 |
| LuCa | Tumor biopsy | *LRRC3B* |  | 30185536 |
| LuCa | Patient-derived xenograft | *LRP12* |  | 30029672 |
| LuCa | Tumor biopsy and Cell Lines | *miR-10b, miR-1179, miR-137, miR-572, miR-3150b, and miR-129-2* |  | 29570800 |
| LuCa | Bronchial biopsy | *HOXA9, SOX17, ZNF154, HBP1, SFRP1, PCDH17, ITGA5* |  | 29270240 |
| LuCa | Tumor biopsy | *ADHFE1, TCF21, TBX5, HNB1* | *TP63, CLCD1* | 29169318 |
| LuCa | Bronchioalveolar lavage | *SHOX2, RASSF1A* |  | 29151944 |
| LuCa | Lung biopsy | *BEND4, ZSCAN31, GPR135* |  | 28722770 |
| LuCa | Sputum | *SOX17, CDO1, ZFP42, TAC1, FAM19A4, FHIT, MGMT, p16, RASSF1A* |  | 28644424 |
| LuCa | Biopsy and serum | *APC, DAPK, GSTP1* |  | 28524770 |
| LuCa | Cell lines | *MLH1* |  | 28214209 |
| LuCa | Cell lines |  | *FTHL17* | 28207785 |
| LuCa | Tumor biopsy and cell lines | *MIR1247* |  | 27942223 |
| LuCa | Buffy Coat | *6p21.33, F2RL3* | *AHRR* | 27924164 |
| LuCa | Sputum | *RASSF1A, PRDM14, 3OST2* |  | 27777637 |
| LuCa | Whole blood | *F2RL3,UGT1A1, NFKBIL1* | *AHRR* | 27503000 |
| LuCa | Cell lines |  | *MALAT1* | 26884862 |
| LuCa | Lymph node, bronchial aspirates, pleural effusion, plasma, and tumor tissue | *SHOX2* |  | 26640383 |
| LuCa | Tumor biopsy |  | *EGFR* | 26505339 |
| LuCa | Tumor biopsy | *SMAD3, SYNPO, GPR88, SND1-IT1, SND1, TMEM212, LOC404266, TTC39C, ZMIZ1, EYA4* | *RUNX1, C22orf9, MIR1249, NTM, CSGAL,NACT1, IPO5, EDARADD, SLAMF8, SLC22A18AS 1, SLC22A18, SMCP, MIR298, MIR296, SCT, PARP4, EPS15, WIPF1, HRH1, FAM49A, CHRM5, RUNX3, TSPAN9, CARD14, S100A3, GNASAS, ZC3H12D, PLEKHA5, LEPR, LEPROT, CHRNA1, ESRRG, STRA6, HTR1D, GSTA3, SH3BP4, LCE1B* | 26449251 |
| LuCa | Tumor biopsy and plasma | *HOXD10, PAX9, PTPRN2, STAG3* |  | 26425700 |
| LuCa | Tumor biopsy and cell lines | *hMLH1* |  | 26191250 |
| LuCa | Lung biopsy | *RASSF1A* |  | 26112163 |
| LuCa | Tumor biopsy and cell lines | *TMEM196* |  | 26056045 |
| LuCa | Cell lines | *DKK3* |  | 25760729 |
| LuCa | Tumor biopsy | *SFTPA2* |  | 25514367 |
| LuCa | Tumor biopsy and cell lines | *AXL, ESRP1, HoxB4, SPINT1/2* |  | 25486910 |
| LuCa | Tumor biopsy | *HIST1H4F, PCDHGB6, NPBWR1, ALX1, and HOXA9.* |  | 24081945 |
| LuCa | Cell lines | *RASSFlA* |  | 24011511 |
| LuCa | Lung biopsy | *VTRNA2-1* |  | 23592755 |
| LuCa | Cell lines | *EGFR* |  | 23440266 |
| LuCa | Bronchioalveolar lavage | *P16, TERT, WT1, RASSF1* |  | 22962272 |
| LuCa | Cell lines | *IL1B, IL6, IL8* |  | 22923190 |
| LuCa | Lung biopsy | *PITX2, SHOX2* |  | 22555092 |
| LuCa | Cell lines | *ERBB2, ZEB2* |  | 22261801 |
| LuCa | Bronchial Aspiration | *SHOX2* |  | 22108652 |
| LuCa | Tumor tissue (focused squamo)Bronchioalveolar lavage | *CCDC37, CYTL1, CD01, LM03, SLIT2* | *SERPINB5* | 22011669 |
| LuCa | Cell lines | *NR3C1* |  | 21984896 |
| LuCa | Bronchial epithelium and sputum | *RASSF1A* |  | 11956099 |
| LuCa | Lung biopsy | *RB1* |  | 23967231 |
| LuCa | Lung biopsy | *APC* |  | 11429699 |
| LuCa | Lung biopsy | *DAPK1, MGMT, RARβ* |  | 1566180 |
| LuCa | Lung biopsy | *FHIT* |  | 27572663 |
| LuCa | Lung biopsy | *GSTP1* |  | 24935385 |
| LuCa | Bronchial aspirates | *SEMA3B* |  | 15849750 |
| LuCa | Lung biopsy | *hOGG1* |  | 20521346 |
| LuCa | Lung Biopsy |  | *MAGE* | 11691819 |
| LuCa | Cell line |  | *SNCG* | 17369845 |
| LuCa | Plasma | *SHOX2* |  | 21694641 |
| LuCa | Tumor biopsy | *SHOX2* |  | 21426551 |
| LuCa | Bronchial aspirate | *SHOX2* |  | 21047392 |
| LuCa | Tumor tissue | *RASSF1, RUNX3* | *LINE-1* | 19764999 |
| LuCa | Serum | *DAPK* |  | 19653236 |
| LuCa | Tumor biopsy | *MTHFR, RASSF1A, CDKN2A* |  | 19118009 |
| LuCa | Tumor biopsy | *GDNF, MTHFR, OPCML, TNFRSF25, TCF21, PAX8, PTPRN2, PITX2.* |  | 18616821 |
| LuCa | Tumor biopsy | *RARB, BVES, CDKN2A, KCNH5, RASSF1, CDH13, RUNX* |  | 18349282 |
| LuCa | Tumor biopsy | *p16, CDH13* |  | 18337602 |
| LuCa | Tumor biopsy | *MEOX2, MDK, LAPTM5, FGFR3* |  | 18203646 |
| LuCa | Tumor biopsy | *OLIG1* |  | 17388669 |
| LuCa | Tumor biopsy | *P16* |  | 17094392 |
| LuCa | Tumor biopsy | *RARbeta, P16* |  | 11196170 |
| Smokers | Small airways | *JAG1,BPIFB1* | *CYP1B1, CYP1A1, ALDH3A1, SFRP2* | 23842454 |
| Smokers | Cell lines | *RASSF1A,RAR-β* | *D4Z4, NBL2,LINE-1* | [20440268](https://www.nature.com/articles/onc2010129) |
| Smokers | Cell lines | *E-cadherin, CDH13, proto-cadherin 10, GATA5, and PAX5* |  | [18974146](https://aacrjournals.org/cancerres/article/68/21/9005/541781/Carcinogen-Induced-Gene-Promoter-Hypermethylation) |
| Smokers | Bronchial brushing | *p16, DAPK* |  | 11809677 |
| Smokers | Oral Mucosal Cells | *PARVA,STXBP5-AS1* | *LINC00673, CYP1B1* | 25517428 |
| Smokers | Peripheral blood - Lymphocytes and macrophages | *MR1, AHRR (* *cg25648203-*  *cg04135110), RFTN1, ZC3H12A* | *AHRR*  *(* *cg14817490-*  *cg05575921-*  *cg14454127-*  *cg03991871)* | 22232023 |
| Smokers | Peripheral blood |  | *AHRR, F2RL3* | [27632354](https://pubmed.ncbi.nlm.nih.gov/27632354/) |
| Smokers | Peripheral blood |  | *CPOX, PTPN6, GNG12* | 24704585 |
| Smokers | Peripheral blood |  | *F2RL3* | [21457905](https://www.sciencedirect.com/science/article/pii/S0002929711000954?via%3Dihub) |
| Smokers | Mononuclear cells | *GPR15 gene (cg19859270)* | *AHRR (cg05575921 and cg23576855)* | 24559495 |
| Smokers | Peripheral blood | *SNED1 , MYO1G , ZIC5,* | *GNG12,GNG12,NOS1AP,AVPR1B,AKT3, 2q37.1 (ALPPL2, APLP, ALPI) , GPR15, CPOX, AHRR, C5orf62 , ZMIZ1, LRP5, ARRB1 ,PRSS23 , C14orf43, ITPK1 , FAM98B, SEMA7A , RARA, F2RL3 , NCF4, BEX4* | 24334605 |
| Smokers | Whole Blood | *HIVEP3, CACNA1D, TIAM2, MYO1G, CNTNAP2, PCDH9* | *EXOSC10-AS1, GNG12, GFI1,*  *ECEL1P2, ALPPL2b, AHRR,*  *CASC8, ZC3H3, LRP5, RARA, LINGO3, F2RL3* | 23691101 |
| Smokers | Sputum - Bronchial epithelial cells | *p16, MGMT, DAP, RASSFIA* |  | 11956099 |
| Smokers | Sputum | *RASSF1A* |  | 12527916 |
| Smokers | Peripheral blood |  | *AHRR, F2RL3* | [26667048](https://www.nature.com/articles/ncomms10192) |
| Smokers | Whole Blood |  | *AHRR, F2RL3* | [32736658](https://clinicalepigeneticsjournal.biomedcentral.com/articles/10.1186/s13148-020-00908-3%20https:) |
| COPD | Small airway epithelium | *GSTP1, CHRNB1, CHRNB2 y CHRND GPR126, HTR4 y EPHX1* | *KSR1* | 24298892 |
| COPD | Peripheral blood | *PIK3CD* |  | 31995399 |
| COPD | Lung tissue | *CCL5,TNFRSF21, SUSD2,* |  | 26349763 |
| COPD | Whole blood | *EPS8L1* |  | 30018765 |
| COPD | Airway epithelium |  | *SPDEF - FOXA2.* | 28450970 |
| COPD | Whole Blood |  | *cg16361890 MAML1, RBFOX2, CD72, GRASP, and SH3TC1.* | 27814717 |
| COPD | Peripheral blood | *RAB4B, CYP2A6, AK097370 NUMBL, EGLN2, DNMT3A, LOC101929709 - PAK2* | *cg11298343-EGLN2* | 29092026 |
| COPD | Peripheral blood | *AREG, E2F1chr20:32274387, HDAC1chr1:32757717* | *ATG3, FOXO3, E2F1 chr20:32273763, NUF2, MMP2, TP53* | 31684967 |
| COPD | Sputum and Peripheral Blood Leukocytes | *ELOVL2, C1orf132, KLF14, TRIM59, and FHL2* |  | 34368190 |
| COPD | Whole blood and lung tissue | *EPAS1* |  | 25569234 |
| COPD | Cellular Lines |  | *NF-κB, STAT3, IKK y NIK* | 32342329 |
| COPD | Lung Tissue | *FRMD4A, THSD4, and C10orf11.* |  | 27564456 |
| COPD | Peripheral blood and epithelial cells. |  | *AHRR* | 36359818 |
| COPD | Whole blood |  | *Global hypomethylation* | 28818709 |
| COPD | Alveolar macrophages |  | *S1PR5* | 27868302 |
| COPD | Airway and parenchymal fibroblasts | *OAT, GRIK2* | *TRPV3* | 29527240 |
| COPD | Polymorphonuclear Cells | *OSBPL5, PRKAG2, GSTM3, F3, TMEM41A, PRKAG2, OSBPL5, MIR1914; UCKL1, cg24867279, PRKAG2, OSBPL5* | *WDR6, ALOX5AP, BST1, WDR6, GAK,PSMD8, cg05129295, FOLR3, WDR6* | 30248490 |
| COPD | Sputum | *CDKN2A, MGMT* |  | 22818553 |

**Supplementary Table S2.** List of Genes with Statistically Significant DNA Methylation Changes across Study Groups.

|  | | **Genes** |
| --- | --- | --- |
| **LUCA** | Hypermethylated  genes | *PCDHGA12,CDO1,SHOX2,RASSF1,CD274,PTGER4,RPTOR,TRPV2,AMPD3,TRAF1,CXXC5,SPON2,DIO3OS,DAPK1,CDKN2A,TAC1,HOXA7,HOXA9,SOX17,ZFP42,MYF6,SIX6,RXRG,LHX1,TERT,MIR9-1, B3GAT2, BCAR1, HLF, HOPX, HOXD11, MIR1203, MYL9, NHERF2, SYT5,VTRNA1-3 ,SPOP, HOXA1, WT1, PITX2, CALCA, CDH13 ,DLEC1, B3GNTL1, F2RL3, DOP1B, KPNA7, KIAA0087, MYO15A, CA5A, NHERF4,CSHL1,MYO9B,CRTAM,OCA2,FES,STXBP6,BCL6B,FZD10,HSPB6,LRRC3B,LRP12,MIR10B,MIR1179,MIR137,MIR572,MIR3150B,MIR1292,ZNF154,HBP1,SFRP1,PCDH17,ITGA5,ADHFE1,TCF21,TBX5,BEND4,ZSCAN31,GPR135,TAFA4,FHIT,MGMT,APC,GSTP1,MLH1,MIR1247,PRDM14,HS3ST2,SMAD3,SYNPO,GPR88,SND1-IT1,SND1,TMEM212,HOXB-AS3, TTC39C, ZMIZ1, EYA4, HOXD10, PAX9, PTPRN2,STAG3,TMEM196,DKK3,SFTPA2,AXL,ESRP1,HOXB4,SPINT1,H4C6,PCDHGB6,NPBWR1,ALX1,VTRNA2-1 , IL1B, IL6, CXCL8, ERBB2, ZEB2, BARHL2, IRX2, MEIS1, MSX1, NR2E1, ONECUT2, OSR1, PAX6, TFAP2A, ZNF577, CFAP100, CYTL1, LMO3, SLIT2, NR3C1, RB1, TGFBR2, CDH1, RARB, SEMA3B, OGG1, ZMYND10, RUNX3, MTHFR, GDNF, OPCML, TNFRSF25, PAX8, BVES, KCNH5, MEOX2, MDK, LAPTM5, FGFR3, OLIG1.* |
|  | Hypomethylated genes | *KIFC1,FUT7,VGLL4,LYPD8,VPS13D,MIR130A,FTHL17,TP63,CLDN1,MALAT1,EGFR,RUNX1,KIAA0930,MIR1249,NTM,CSGALNACT1,IPO5,EDARADD,SLAMF8,SLC22A18AS,SLC22A18,SMCP,MIR298,MIR296,SCT,PARP4,EPS15,WIPF1,HRH1,CYRIA,CHRM5,TSPAN9,CARD14,S100A3,GNASAS1, ZC3H12D, PLEKHA5, LEPR, LEPROT, CHRNA1, ESRRG, STRA6, HTR1D, GSTA3, SH3BP4, LCE1B, SERPINB5, MAGEA1, SNCG, L1TD1,AHRR* |
| **SMOKERS** | Hypermethylated genes | *JAG1,BPIFB1,RASSF1,RARB,CHD1,CDH13,PCDH10,GATA5,PAX5,CDKN2A,DAPK1,PARVA,STXBP5-AS1,MR1,RFTN1,ZC3H12A,SNED1,MYO1G,ZIC5,HIVEP3,CACNA1D,TIAM2,CNTNAP2,PCDH9,MGMT,MTNR1A,KRT14,KRT19,MIR9-3,MIR137* |
|  | Hypomethylated genes | *CYP1B1,CYP1A1,ALDH3A1,SFRP2,DUX4,DUX4L8,L1TD1,LINC00511,AHRR,F2RL3,CPOX,PTPN6,GNG12,NOS1AP,AVPR1B,AKT3,ALPG,ALPI,SMIM3,ZMIZ1,LRP5,ARRB1,PRSS23,MIDEAS,ITPK1,FAM98B,SEMA7A,RARA,NCF4,BEX4,GFI1,ZC3H3,LINGO3,GPR15* |
| **COPD** | Hypermethylated genes | *GSTP1,CHRNB1,CHRNB2,CHRND,ADGRG6,HTR4,EPHX1,PIK3CD,CCL5,TNFRSF21,SUSD2,RAB4B,CYP2A6,CYP2T1P,NUMBL,DNMT3A,PARAIL,PAK2,AREG,E2F1,HDAC1,ELOVL2,MIR29B2CHG,KLF14,TRIM59,FHL2,EPAS1,FRMD4A,THSD4,LRMDA,OAT,GRIK2,OSBPL5,PRKAG2,GSTM3,F3,TMEM41A,MIR1914,UCKL1* |
|  | Hypomethylated genes | *KSR1,SPDEF,FOXA2,MAML1,RBFOX2,CD72,TAMALIN,SH3TC1,EGLN2,ATG3,FOXO3,NUF2,MMP2,TP53,NFKB1,STAT3,IKBKB,MAP3K14,S1PR5,TRPV3,WDR6,ALOX5AP,BST1,GAK,PSMD8,FOLR3,AHRR* |

**Supplementary Table S3.** Common biological processes and signaling pathways associated with differentially methylated Genes in Lung Cancer, COPD, and Smoking.

| Term | Category | LuCa | Smokers | COPD |
| --- | --- | --- | --- | --- |
| Signal transduction | Biological process  (GOTERM_BP_DIRECT) | *AXL, BCAR1, CXCL8, CD274, EDARADD, F2RL3, LRP12, RASSF1A, TAFA4, TNFRSF25, CHRNA1, CYTL1, DAPK1, EGFR, ERBB2, GDNF, IL1B, MDK, NR3C1, RARB, SCT* | *AKT3, F2RL3, GNG12, RASSF1A, ARRB1, DAPK1, ITPK1, RARA, RARB* | *TNFRSF21, BST1, CHRNB1, CHRNB2, CHRND, EPAS1, KSR1, PAK2, PIK3CD, STAT3, TAMALIN* |
| Regulation of transcription from RNA polymerase II promoter | Biological process  (GOTERM_BP_DIRECT) | *ALX1, BCL6B, BARHL2, HLF, HBP1, HOPX, LHX1, MEIS1, PRDM14, RB1, RUNX1, RUNX3, SHOX2, SIX6, SMAD3,SOX17, TBX5, WT1, ZFP42, AHRR, ESRRG, HOXA1, HOXA7, HOXA9, HOXB4, HOXD10, HOXD11, IRX2, MEOX2, MSX1, MYF6, NR2E1, NR3C1, OSR1,OLIG1,ONECUT2,PAX6, PAX8, PAX9, PITX2, RXRG, TCF21, TFAP2A, TP63, ZEB2, ZMIZ1, ZSCAN31, ZNF154, ZNF577* | *HIVEP3, ZIC5, ARRB1, AHRR, CHD1, DUX4, GFI1, MIDEAS, PAX5, ZMIZ1* | *E2F1, KLF14, SPDEF, AHRR, EPAS1, FOXA2, FOXO3, HDAC1, NFKB1, STAT3, TP53* |
| Positive regulation of angiogenesis | Biological process  (GOTERM_BP_DIRECT) | *CXCL8, RUNX1, HSPB6, IL1B, MIR130A, NR2E1, TERT, TGFBR2* | *AKT3, CYP1B1, SFRP2, ZC3H12A* | *F3, PIK3CD, STAT3* |
| Positive regulation of gene expression | Biological process  (GOTERM_BP_DIRECT) | *CXCL8, SMAD3, SOX17, WT1, CDKN2A, ERBB2, IL1B, IL6, KPNA7, MIR130A, OSR1, PAX6, TFAP2A* | *GATA5, CDKN2A, FAM98B, NOS1AP, RARA, ZC3H12A* | *E2F1, F3, HDAC1, PIK3CD, STAT3, TP53* |
| Positive regulation of transcription from RNA polymerase II promoter | Biological process  (GOTERM_BP_DIRECT) | *RB1, BARHL2, HLF, ONECUT2, TCF21, NR2E1, NR3C1, MEOX2, HOXD10, EGFR, HOXA9, SOX17, ZMIZ1, CYTL1, ALX1, HOXA1, MSX1, HOXA7, PITX2, RXRG, TP63, TFAP2A, SMAD3, CDKN2A, OSR1, LMO3, PAX6, OLIG1, ESRRG, TBX5, RUNX1, ZEB2, IL6, MEIS1, PAX8, GDNF, WT1, IL1B, PAX9, MYF6, HOXB4, RARB, CDH13* | *GATA5, LRP5, ARRB1, CDH13, CDKN2A, DUX4, JAG1, PAX5, RARA, RARB, SFRP2, ZC3H12A, ZMIZ1* | *E2F1, KLF14, SPDEF, EPAS1, FOXA2, FOXO3, HDAC1, IKBKB, MAML1, NFKB1, STAT3, TP53* |
| Positive regulation of apoptotic process | Biological process  (GOTERM_BP_DIRECT) | *MIR137, IL6, SFRP1, APC, WT1, CDKN2A, DAPK1, RARB, SLIT2* | *CDKN2A, CYP1B1, DAPK1, MIR137, RARB, SFRP2* | *E2F1, SPDEF, FOXO3, MMP2, TP53* |
| Pathways in cancer | KEGG_PATHWAY | *APC, CXCL8, F2RL3, RB1, RUNX1, RASSF1A, SMAD3, TRAF1, CDH1, CDKN2A, DAPK1, EGFR, ERBB2, FGFR3 , FZD10, GSTA3, GSTP1, IL6, MLH1, PAX8, PTGER4, RARB, RXRG, TERT, TGFBR2* | *AKT3,F2RL3, GNG12, LRP5, RASSF1A, CDKN2A, DAPK1, JAG1, RARA, RARB* | *E2F1, EGLN2, EPAS1, GSTM3, GSTP1, HDAC1, IKBKB, MMP2, NFKB1, PIK3CD, STAT3, TP53* |
| Bladder cancer | KEGG_PATHWAY | *CXCL8, RB1, RASSF1A, CDH1, CDKN2A, DAPK1, EGFR, ERBB2,FGFR3* | *RASSF1A, CDKN2A, DAPK1* | *E2F1, MMP2, TP53* |
| Non-small cell lung cancer | KEGG_PATHWAY | *RB1, RASSF1A, CDKN2A, ERBB2, RARB, FHIT, RXRG, EGFR* | *AKT3, RASSF1A, CDKN2A, RARB* | *E2F1, FOXO3, PIK3CD, STAT3, TP53* |
| MicroRNAs in cancer | KEGG_PATHWAY | *MIR10B, CDKN2A, MIR9-1, HOXD10, EGFR, SERPINB5, RPTOR, MIR137, ZEB2, RASSF1A, APC, ERBB2, ITGA5, FGFR3, TP63, MIR129-2* | *RASSF1A, CDKN2A, CYP1B1, MIR137, MIR9-3* | *DNMT3A, E2F1, HDAC1, IKBKB, NFKB1, PIK3CD, STAT3, TP53* |

**Supplementary Table S4.** Pathways in Cancer Associated with Differentially Methylated Genes Identified Using KEGG.

| **Pathway map** | **Pathways in cancer - Name** | **Differentially methylated genes** | **Process** |
| --- | --- | --- | --- |
| **LuCa** | | | |
| [map04520](https://www.kegg.jp/entry/map04520) | Cell-cell adherents junctions (AJs) | *CDH1* | Tissue invasion and metastasis |
| [map04310](https://www.kegg.jp/entry/map04310) | Wnt signaling pathway | *FZD10, APC, F2RL3, PTGER4* | Tissue invasion and metastasis |
| [map04060](https://www.kegg.jp/entry/map04060) | Cytokine-cytokine receptor interaction | *IL6* | Sustained angiogenesis |
| [map04010](https://www.kegg.jp/entry/map04010) | MAPK signaling pathway | *EGFR, ERBB2, FGFR3, RASSF1, DAPK1, CXCL8* | Proliferation and Sustained angiogenesis |
| [map04020](https://www.kegg.jp/entry/map04020) | Calcium signaling pathway | *RASSF1* | Proliferation, Evading apoptosis, and Sustained angiogenesis |
| [map03320](https://www.kegg.jp/entry/map03320) | PPAR signaling pathway | *PAX8, RXRG* | Proliferation |
| [map07223](https://www.kegg.jp/entry/map07223) | Retinoic acid receptor (RAR) and retinoid X receptor (RXR) agonists/antagonists | *RARB, RXRG* | Proliferation |
| [map04915](https://www.kegg.jp/entry/map04915) | Estrogen signaling pathway | *RUNX1* | Proliferation |
| [map04110](https://www.kegg.jp/entry/map04110) | Cell Cycle | *RB1, CDKN2A, TERT* | Proliferation |
| [map05208](https://www.kegg.jp/entry/map05208) | Chemical carcinogenesis - reactive oxygen species | *GSTP1, GSTA3* | Proliferation and Evading apoptosis |
| [map04350](https://www.kegg.jp/entry/map04350) | TGF-beta signaling pathway | *TGFBR2, SMAD3, RUNX1, MLH1* | Insensitivity to anti-growth signals |
| [map04330](https://www.kegg.jp/entry/map04330) | Notch signaling pathway | *ERBB2* | Sustained angiogenesis |
| [map04064](https://www.kegg.jp/entry/map04064) | NF-kappa B signaling pathway | *TRAF1* | Evading apoptosis |
| **Smokers** | | | |
| [map04330](https://www.kegg.jp/entry/map04330) | Notch signaling pathway | *JAG1* | Sustained angiogenesis |
| [map04310](https://www.kegg.jp/entry/map04310) | Wnt signaling pathway | *LRP5* | Evading apoptosis, Proliferation |
| [map04151](https://www.kegg.jp/entry/map04151) | PI3K-Akt signaling pathway | *AKT3, F2RL3, GNG12* | Evading apoptosis, Proliferation |
| [map04150](https://www.kegg.jp/entry/map04150) | mTOR signaling pathway | *AKT3* | Regulation of actin cytoskeleton |
| [map04014](https://www.kegg.jp/entry/map04014) | Ras signaling pathway | *RASSF1, GNG12* | Sustained angiogenesis, Proliferation |
| [map04010](https://www.kegg.jp/entry/map04010) | MAPK signaling pathway | *DAPK1* | Sustained angiogenesis, Proliferation |
| [map07223](https://www.kegg.jp/entry/map07223) | Retinoic acid receptor (RAR) and retinoid X receptor (RXR) agonists/antagonists | *RARB* | Proliferation |
| [map04915](https://www.kegg.jp/entry/map04915) | Estrogen signaling pathway | *RARA* | Proliferation |
| [map04115](https://www.kegg.jp/entry/map04115) | p53 signaling pathway | *CDKN2A* | Proliferation, Evading apoptosis |
| [map04110](https://www.kegg.jp/entry/map04110) | Cell Cycle | *CDKN2A* | Proliferation |
| **COPD** | | | |
| [map04066](https://www.kegg.jp/entry/map04066) | HIF-1 signaling pathway | *EGLN2, EPAS1* | Sustained angiogenesis |
| [map04151](https://www.kegg.jp/entry/map04151) | PI3K-Akt signaling pathway | *PIK3CD, IKBKB, NFKB1, TP53* | Evading apoptosis, Proliferation, Sustained angiogenesis |
| [map04630](https://www.kegg.jp/entry/map04630) | JAK-STAT signaling pathway | *STAT3* | Evading apoptosis |
| [map04060](https://www.kegg.jp/entry/map04060) | Cytokine-cytokine receptor interaction | *STAT3* | Sustained angiogenesis |
| [map04010](https://www.kegg.jp/entry/map04010) | MAPK signaling pathway | *MMP2* | Sustained angiogenesis, Proliferation |
| [map04915](https://www.kegg.jp/entry/map04915) | Estrogen signaling pathway | *E2F1* | Block of differentiation |
| [map04110](https://www.kegg.jp/entry/map04110) | Cell Cycle | *E2F1* | Proliferation |
| [map04115](https://www.kegg.jp/entry/map04115) | p53 signaling pathway | *TP53* | Proliferation, Evading apoptosis |
| [map05204](https://www.kegg.jp/entry/map05204) | Chemical carcinogenesis - DNA adducts | *GSTP1* | Genomic damage |
| [map04350](https://www.kegg.jp/entry/map04350) | TGF-beta signaling pathway | *HDAC1* | Insensitivity to anti-growth signals |
| [map05208](https://www.kegg.jp/entry/map05208) | Chemical carcinogenesis - reactive oxygen species | *GSTM3, GSTP1* | Proliferation, Evading apoptosis |

**Supplementary Table S5.** Identify transcription factor binding sites (TFBS) in differential methylation genes.

| **Transcription Factor** | **Motif** | **Adjusted**  ***p-value*** | **Intersection** | **Genes** |
| --- | --- | --- | --- | --- |
| MAZ | GGGGGAGGGGGNGRGRRRGNRG | 5,99E-05 | 98/135 | *ALX1,APC,AXL,BARHL2,BCAR1,BCL6B,CDH1,CDKN2A,CYTL1,EDARADD,EGFR,ERBB2,ESRRG,F2RL3,FGFR3,FHIT,FZD10,GDNF,GSTP1,HBP1,HLF,HOPX,HOXA1,HOXA7,HOXB4,HOXD11,IRX2,ITGA5,LHX1,LMO3,LRP12,MDK,MSX1,MYF6,NR2E1,OLIG1,ONECUT2,OSR1,PAX9,PITX2,PTGER4,RPTOR,RUNX1,RXRG,SCT,SERPINB5,SFRP1,SHOX2,SIX6,SMAD3,SOX17,TAFA4,TERT,TFAP2A,TNFRSF25,WT1,ZEB2,ZMIZ1,AKT3,ARRB1,CHD1,CYP1B1,GATA5,GFI1,GNG12,ITPK1,JAG1,LRP5,MIDEAS,NOS1AP,PAX5,RARA,SFRP2,ZC3H12A,ZIC5,BST1,CHRNB1,CHRNB2,CHRND,DNMT3A,E2F1,EGLN2,EPAS1,F3,FOXA2,FOXO3,GSTM3,HDAC1,IKBKB,KLF14,KSR1,MMP2,NFKB1,PAK2,PIK3CD,STAT3,TAMALIN,TP53* |
| AP-2alpha | NGCCYSNNGSN | 9,10E-05 | 95/135 | *ALX1,BARHL2,BCAR1,BCL6B,CDH13,CDKN2A,CHRNA1,CYTL1,EDARADD,EGFR,ERBB2,F2RL3,FGFR3,FZD10,GDNF,GSTP1,HBP1,HLF,HOXA1,HOXA7,HOXB4,HOXD11,HSPB6,IL6,IRX2,ITGA5,KPNA7,LHX1,MDK,MEOX2,MSX1,MYF6,OLIG1,ONECUT2,OSR1,PAX8,PAX9,PITX2,PRDM14,PTGER4,RB1,RUNX1,RUNX3,SCT,SERPINB5,SFRP1,SIX6,SLIT2,SMAD3,SOX17,TBX5,TERT,TGFBR2,TNFRSF25,TRAF1,WT1,ZEB2,ZMIZ1,AKT3,ARRB1,CHD1,CYP1B1,GATA5,GFI1,GNG12,ITPK1,JAG1,LRP5,NOS1AP,PAX5,RARA,ZC3H12A,ZIC5,CHRNB1,CHRND,DNMT3A,E2F1,EGLN2,EPAS1,F3,FOXA2,FOXO3,GSTM3,HDAC1,IKBKB,KLF14,KSR1,MMP2,NFKB1,PAK2,PIK3CD,STAT3,TAMALIN,TNFRSF21,TP53* |
| ZNF148 | NNCCCCTCCCCC | 2,13E-04 | 78/135 | *AHRR,APC,AXL,BCAR1,CDH1,CDKN2A,EDARADD,ERBB2,F2RL3,FZD10,GDNF,GSTP1,HBP1,HLF,HOPX,HOXA1,HOXA7,HOXB4,HOXD11,HSPB6,IL1B,IL6,IRX2,ITGA5,LHX1,LRP12,MDK,MSX1,MYF6,NR2E1,OLIG1,ONECUT2,OSR1,PAX8,PAX9,PRDM14,RARB,RPTOR,SCT,SHOX2,SIX6,SOX17,TERT,TFAP2A,WT1,ZEB2,ZNF577,AKT3,ARRB1,CHD1,CYP1B1,GATA5,GNG12,JAG1,LRP5,MIDEAS,NOS1AP,RARA,SFRP2,ZC3H12A,ZIC5,BST1,CHRNB1,CHRNB2,DNMT3A,E2F1,EGLN2,FOXA2,FOXO3,GSTM3,HDAC1,IKBKB,KLF14,KSR1,MMP2,NFKB1,PAK2,PIK3CD* |
| ETF | CCCCGCCCCYN | 1,08E-03 | 119/135 | *AHRR,ALX1,APC,AXL,BARHL2,BCAR1,BCL6B,CDH1,CDKN2A,CYTL1,DAPK1,EDARADD,EGFR,ERBB2,ESRRG,F2RL3,FGFR3,FHIT,FZD10,GDNF,GSTA3,GSTP1,HBP1,HLF,HOXA1,HOXA7,HOXA9,HOXB4,HOXD11,HSPB6,IRX2,ITGA5,KPNA7,LHX1,LMO3,LRP12,MDK,MEOX2,MSX1,MYF6,NR2E1,OLIG1,ONECUT2,OSR1,PAX6,PAX9,PITX2,PRDM14,PTGER4,RARB,RASSF1,RB1,RPTOR,RUNX1,RUNX3,RXRG,SCT,SERPINB5,SFRP1,SHOX2,SIX6,SLIT2,SMAD3,SOX17,TAFA4,TBX5,TERT,TFAP2A,TGFBR2,TNFRSF25,TRAF1,WT1,ZEB2,ZMIZ1,ZNF154,ZNF577,AKT3,ARRB1,CHD1,CYP1B1,FAM98B,GATA5,GFI1,GNG12,ITPK1,JAG1,LRP5,MIDEAS,NOS1AP,PAX5,RARA,SFRP2,ZC3H12A,ZIC5,BST1,CHRNB1,CHRNB2,CHRND,DNMT3A,E2F1,EGLN2,EPAS1,F3,FOXA2,FOXO3,GSTM3,HDAC1,IKBKB,KLF14,KSR1,MMP2,NFKB1,PAK2,PIK3CD,SPDEF,STAT3,TAMALIN,TNFRSF21,TP53* |
| Churchill | CGGGNN | 1,69E-03 | 100/135 | *AHRR,APC,BARHL2,BCAR1,BCL6B,CDH1,CDH13,CDKN2A,CHRNA1,CYTL1,DAPK1,EDARADD,EGFR,ERBB2,ESRRG,F2RL3,FGFR3,FZD10,GDNF,GSTP1,HBP1,HLF,HOXA1,HOXA7,HOXB4,HOXD11,HSPB6,IL6,IRX2,ITGA5,LRP12,MDK,MLH1,MSX1,MYF6,OLIG1,ONECUT2,PAX8,PITX2,PRDM14,RASSF1,RB1,RUNX1,SCT,SFRP1,SHOX2,SLIT2,SMAD3,SOX17,TAFA4,TBX5,TCF21,TERT,TFAP2A,TGFBR2,TNFRSF25,TRAF1,WT1,ZEB2,ZMIZ1,AKT3,ARRB1,CHD1,CYP1B1,GATA5,GFI1,GNG12,HIVEP3,ITPK1,JAG1,LRP5,MIDEAS,NOS1AP,PAX5,RARA,SFRP2,ZC3H12A,ZIC5,CHRNB1,CHRNB2,CHRND,DNMT3A,E2F1,EGLN2,EPAS1,F3,FOXA2,FOXO3,HDAC1,IKBKB,KLF14,KSR1,MMP2,NFKB1,PIK3CD,SPDEF,STAT3,TAMALIN,TNFRSF21,TP53* |
| ZNF253 | SNGNSCGNGGNGCKGNN | 3,55E-03 | 73/135 | *AXL,BARHL2,BCAR1,BCL6B,DAPK1,EDARADD,EGFR,ERBB2,F2RL3,FGFR3,FZD10,GDNF,GSTP1,HLF,HOXA1,HOXA7,HOXA9,HOXD11,IRX2,ITGA5,MDK,MEOX2,MSX1,OLIG1,ONECUT2,PAX9,PRDM14,RUNX1,SCT,SFRP1,SMAD3,SOX17,TCF21,TERT,TFAP2A,TGFBR2,TNFRSF25,TRAF1,WT1,ZEB2,ZMIZ1,AKT3,ARRB1,CHD1,CYP1B1,GATA5,GFI1,GNG12,ITPK1,JAG1,LRP5,MIDEAS,NOS1AP,SFRP2,ZC3H12A,ZIC5,BST1,CHRNB1,DNMT3A,E2F1,EGLN2,EPAS1,F3,FOXA2,FOXO3,IKBKB,KLF14,KSR1,NFKB1,PAK2,PIK3CD,TAMALIN,TNFRSF21* |
| ZNF37A | CCYYGGCTCCNTSCCMN | 4,33E-03 | 102/135 | *AHRR,ALX1,APC,AXL,BARHL2,BCAR1,BCL6B,CDKN2A,CYTL1,DAPK1,EDARADD,EGFR,ERBB2,F2RL3,FGFR3,FHIT,FZD10,GDNF,GSTP1,HBP1,HLF,HOXA1,HOXA9,HOXD10,HOXD11,HSPB6,IL6,IRX2,ITGA5,KPNA7,LHX1,LMO3,LRP12,MDK,MSX1,NR3C1,OLIG1,ONECUT2,OSR1,PAX6,PAX8,PITX2,PRDM14,PTGER4,RARB,RASSF1,RB1,RXRG,SCT,SERPINB5,SFRP1,SIX6,SLIT2,SMAD3,SOX17,TBX5,TCF21,TERT,TFAP2A,TGFBR2,TNFRSF25,TRAF1,WT1,ZEB2,ZMIZ1,ZNF154,AKT3,CHD1,CYP1B1,FAM98B,GATA5,GFI1,GNG12,HIVEP3,ITPK1,JAG1,LRP5,NOS1AP,PAX5,RARA,SFRP2,ZC3H12A,ZIC5,CHRNB1,CHRNB2,CHRND,DNMT3A,E2F1,EGLN2,EPAS1,F3,FOXA2,FOXO3,GSTM3,HDAC1,IKBKB,KLF14,KSR1,MMP2,NFKB1,PIK3CD,SPDEF* |
| MED8 | CYYNSCYYCCTSCNCC | 6,27E-03 | 81/135 | *ALX1,APC,AXL,BARHL2,CDKN2A,CHRNA1,DAPK1,EDARADD,EGFR,ERBB2,FGFR3,FHIT,FZD10,GDNF,GSTP1,HBP1,HLF,HOPX,HOXA1,HOXA9,HOXB4,HOXD10,HOXD11,HSPB6,IRX2,ITGA5,LHX1,LRP12,MDK,MSX1,NR2E1,OLIG1,ONECUT2,OSR1,PAX6,PITX2,PTGER4,RB1,RPTOR,SCT,SFRP1,SHOX2,SIX6,SMAD3,SOX17,TAFA4,TFAP2A,WT1,ZEB2,ZMIZ1,AKT3,ARRB1,CHD1,GATA5,GNG12,HIVEP3,ITPK1,JAG1,LRP5,MIDEAS,NOS1AP,PAX5,RARA,SFRP2,ZC3H12A,ZIC5,CHRNB2,DNMT3A,EGLN2,FOXA2,FOXO3,HDAC1,IKBKB,KLF14,KSR1,MMP2,NFKB1,PAK2,PIK3CD,STAT3,TAMALIN* |
| E2F-2 | GCGCGCGCGYW | 9,47E-03 | 110/135 | *AHRR,ALX1,BARHL2,BCAR1,BCL6B,CDH1,CDH13,CDKN2A,DAPK1,EDARADD,EGFR,ERBB2,FGFR3,FHIT,FZD10,GDNF,GSTP1,HBP1,HLF,HOXA1,HOXA7,HOXA9,HOXB4,HOXD10,HOXD11,HSPB6,IL6,IRX2,ITGA5,LHX1,LRP12,MDK,MEOX2,MLH1,MSX1,NR2E1,OLIG1,ONECUT2,OSR1,PAX6,PAX8,PAX9,PITX2,PRDM14,PTGER4,RARB,RASSF1,RB1,RPTOR,RUNX1,SCT,SFRP1,SHOX2,SIX6,SLIT2,SMAD3,SOX17,TAFA4,TBX5,TCF21,TERT,TFAP2A,TGFBR2,TNFRSF25,TRAF1,WT1,ZEB2,ZFP42,ZMIZ1,ZNF154,ZNF577,AKT3,ARRB1,CHD1,CYP1B1,FAM98B,GATA5,GFI1,GNG12,HIVEP3,ITPK1,JAG1,LRP5,MIDEAS,NOS1AP,PAX5,RARA,SFRP2,ZC3H12A,ZIC5,BST1,CHRNB1,CHRNB2,DNMT3A,E2F1,EGLN2,EPAS1,F3,FOXA2,FOXO3,GSTM3,IKBKB,KLF14,KSR1,MMP2,NFKB1,PAK2,PIK3CD,TAMALIN,TNFRSF21* |
| WT1 | RGGNGGGGGAGGRGGNGGRG | 1,05E+16 | 71/135 | *ALX1,APC,AXL,BARHL2,BCL6B,CDH1,CDKN2A,CYTL1,EDARADD,EGFR,ERBB2,F2RL3,FGFR3,FZD10,GDNF,GSTP1,HBP1,HLF,HOXA7,HOXB4,HOXD11,IL1B,IL6,IRX2,ITGA5,LHX1,LRP12,MSX1,MYF6,OLIG1,ONECUT2,OSR1,PITX2,RARB,RB1,RPTOR,RXRG,SCT,SHOX2,SIX6,SMAD3,SOX17,TAFA4,TFAP2A,TNFRSF25,ZEB2,AKT3,ARRB1,CHD1,CYP1B1,GNG12,ITPK1,JAG1,LRP5,MIDEAS,NOS1AP,RARA,ZIC5,CHRNB2,CHRND,DNMT3A,E2F1,EGLN2,EPAS1,FOXA2,HDAC1,KSR1,MMP2,NFKB1,PAK2,PIK3CD* |
| ZXDL | GSGSCNNGGGMRGCNCCGGGS | 1,09E+16 | 72/135 | *AHRR,AXL,BARHL2,BCAR1,CDKN2A,DAPK1,EDARADD,EGFR,ERBB2,FGFR3,FZD10,GDNF,HOXA9,HOXB4,HOXD11,HSPB6,IRX2,ITGA5,LHX1,LRP12,MDK,MSX1,ONECUT2,PRDM14,PTGER4,RB1,RUNX1,RXRG,SCT,SFRP1,SHOX2,SLIT2,SMAD3,TBX5,TERT,TFAP2A,TNFRSF25,TRAF1,WT1,ZEB2,ZMIZ1,AKT3,ARRB1,CHD1,CYP1B1,GATA5,GFI1,GNG12,HIVEP3,ITPK1,JAG1,LRP5,MIDEAS,NOS1AP,ZC3H12A,ZIC5,CHRNB1,DNMT3A,E2F1,EGLN2,EPAS1,F3,FOXO3,GSTM3,HDAC1,KLF14,KSR1,MMP2,NFKB1,PIK3CD,TAMALIN,TNFRSF21* |
| AP-2 | MKCCCSCNGGCG | 1,26E+16 | 99/135 | *AHRR,AXL,BARHL2,BCAR1,CD274,CDH1,CDH13,CDKN2A,DAPK1,EDARADD,EGFR,ERBB2,ESRRG,F2RL3,FGFR3,FHIT,FZD10,GDNF,GSTP1,HLF,HOXA1,HOXA7,HOXA9,HOXB4,HOXD11,HSPB6,IL6,IRX2,ITGA5,LHX1,LRP12,MDK,MSX1,MYF6,NR2E1,OLIG1,ONECUT2,OSR1,PAX6,PAX9,PITX2,PRDM14,PTGER4,RASSF1,RB1,RPTOR,RUNX1,RUNX3,SCT,SFRP1,SHOX2,SIX6,SLIT2,SMAD3,SOX17,TAFA4,TERT,TGFBR2,TNFRSF25,TRAF1,WT1,ZEB2,ZMIZ1,ZNF154,AKT3,ARRB1,CHD1,CYP1B1,FAM98B,GFI1,GNG12,ITPK1,JAG1,LRP5,MIDEAS,NOS1AP,RARA,SFRP2,ZIC5,CHRNB1,CHRNB2,CHRND,DNMT3A,E2F1,EGLN2,EPAS1,F3,FOXA2,FOXO3,GSTM3,IKBKB,KLF14,KSR1,MMP2,PAK2,PIK3CD,STAT3,TAMALIN,TNFRSF21* |
| ZF5 | NRNGNGCGCGCWN | 1,42E+16 | 110/135 | *AHRR,ALX1,APC,BARHL2,BCAR1,BCL6B,CDH1,CDH13,DAPK1,EDARADD,EGFR,ERBB2,F2RL3,FGFR3,FZD10,GDNF,GSTP1,HBP1,HLF,HOXA1,HOXA7,HOXA9,HOXB4,HOXD10,HOXD11,HSPB6,IRX2,ITGA5,LHX1,LRP12,MDK,MEOX2,MLH1,MSX1,NR2E1,OLIG1,ONECUT2,OSR1,PAX6,PAX8,PAX9,PITX2,PRDM14,PTGER4,RARB,RASSF1,RB1,RPTOR,RUNX1,RUNX3,SCT,SFRP1,SHOX2,SIX6,SLIT2,SMAD3,SOX17,TAFA4,TBX5,TCF21,TERT,TFAP2A,TGFBR2,TNFRSF25,TRAF1,WT1,ZEB2,ZMIZ1,ZNF154,ZNF577,AKT3,ARRB1,CHD1,CYP1B1,FAM98B,GATA5,GFI1,GNG12,HIVEP3,ITPK1,JAG1,LRP5,MIDEAS,NOS1AP,PAX5,RARA,SFRP2,ZC3H12A,ZIC5,BST1,CHRNB1,CHRND,DNMT3A,E2F1,EGLN2,EPAS1,F3,FOXA2,FOXO3,IKBKB,KLF14,KSR1,MMP2,NFKB1,PAK2,PIK3CD,SPDEF,STAT3,TAMALIN,TNFRSF21* |
| ZNF219 | SNNCAGCACCNNGGNCAGCGSC | 1,77E+15 | 71/135 | *AHRR,BARHL2,BCAR1,BCL6B,CDH1,CDH13,DAPK1,EGFR,ERBB2,F2RL3,FGFR3,FZD10,GDNF,GSTP1,HOXA7,HOXD11,HSPB6,IRX2,ITGA5,LHX1,MDK,MEOX2,OLIG1,ONECUT2,OSR1,PAX9,PITX2,PRDM14,RUNX1,SCT,SFRP1,SHOX2,SLIT2,SMAD3,TCF21,TERT,TFAP2A,TGFBR2,TRAF1,WT1,ZEB2,ZMIZ1,AKT3,ARRB1,CHD1,GATA5,GNG12,ITPK1,JAG1,LRP5,MIDEAS,NOS1AP,PAX5,SFRP2,ZC3H12A,ZIC5,BST1,CHRNB2,CHRND,EGLN2,EPAS1,F3,FOXO3,GSTM3,KLF14,KSR1,NFKB1,PAK2,PIK3CD,TAMALIN,TP53* |
| GKLF | NNRRGRRNGNSNNN | 2,33E+15 | 82/135 | *ALX1,APC,AXL,BCAR1,BCL6B,CDH1,CYTL1,DAPK1,ERBB2,F2RL3,FZD10,GDNF,GSTP1,HBP1,HLF,HOXA1,HOXA7,HOXA9,HOXB4,HOXD11,HSPB6,IL6,IRX2,ITGA5,KPNA7,LHX1,LRP12,MDK,MSX1,NR2E1,OLIG1,ONECUT2,OSR1,PAX8,PAX9,PRDM14,RB1,RPTOR,RXRG,SCT,SHOX2,SIX6,SLIT2,SMAD3,TBX5,TERT,TFAP2A,TNFRSF25,WT1,ZEB2,ZMIZ1,AKT3,CHD1,GATA5,GFI1,JAG1,LRP5,MIDEAS,NOS1AP,RARA,SFRP2,ZC3H12A,ZIC5,BST1,CHRNB2,CHRND,DNMT3A,EGLN2,FOXA2,FOXO3,GSTM3,HDAC1,IKBKB,KLF14,KSR1,MMP2,NFKB1,PAK2,PIK3CD,STAT3,TAMALIN,TNFRSF21* |
| EGR | CGCCCCCGCNN | 2,50E+15 | 43/135 | *BARHL2,BCAR1,CDH1,CDKN2A,EGFR,FGFR3,FZD10,HBP1,HOXA1,HOXA7,HOXA9,HOXD11,IRX2,ITGA5,LHX1,LRP12,MDK,ONECUT2,RUNX1,SCT,SMAD3,TAFA4,TERT,TNFRSF25,ZEB2,ZMIZ1,AKT3,ARRB1,CHD1,GFI1,GNG12,ITPK1,RARA,SFRP2,ZC3H12A,ZIC5,EGLN2,EPAS1,FOXA2,HDAC1,KLF14,KSR1,SPDEF* |
| Egr-1 | GCGGGGGCGG | 2,61E+16 | 80/135 | *AHRR,AXL,BARHL2,BCAR1,BCL6B,CDH1,CDKN2A,EGFR,ERBB2,ESRRG,F2RL3,FGFR3,FZD10,GSTP1,HBP1,HLF,HOXA1,HOXA7,HOXA9,HOXB4,HOXD10,HOXD11,HSPB6,IRX2,ITGA5,LHX1,LRP12,MDK,MSX1,OLIG1,ONECUT2,PITX2,PRDM14,RB1,RUNX1,RUNX3,SCT,SFRP1,SHOX2,SLIT2,SMAD3,TAFA4,TERT,TNFRSF25,ZEB2,ZMIZ1,AKT3,ARRB1,CHD1,GATA5,GFI1,GNG12,ITPK1,JAG1,LRP5,MIDEAS,NOS1AP,PAX5,RARA,SFRP2,ZC3H12A,ZIC5,CHRNB1,DNMT3A,E2F1,EGLN2,EPAS1,F3,FOXA2,FOXO3,HDAC1,IKBKB,KLF14,KSR1,MMP2,NFKB1,PIK3CD,SPDEF,TAMALIN,TP53* |
| MOVO-B | GNGGGGG | 2,62E+16 | 96/135 | *AHRR,APC,AXL,BARHL2,BCAR1,BCL6B,CDH1,CDH13,CDKN2A,CYTL1,DAPK1,EDARADD,EGFR,ERBB2,ESRRG,F2RL3,FGFR3,FZD10,GDNF,GSTP1,HBP1,HLF,HOXA1,HOXA7,HOXB4,HOXD10,HOXD11,HSPB6,IRX2,ITGA5,LRP12,MDK,MLH1,MSX1,ONECUT2,OSR1,PAX8,PITX2,PRDM14,PTGER4,RARB,RASSF1,RB1,RUNX1,SCT,SFRP1,SHOX2,SMAD3,TAFA4,TBX5,TCF21,TERT,TGFBR2,TNFRSF25,TRAF1,WT1,ZEB2,ZMIZ1,AKT3,ARRB1,CHD1,CYP1B1,GATA5,GFI1,GNG12,HIVEP3,ITPK1,JAG1,LRP5,MIDEAS,NOS1AP,PAX5,RARA,ZC3H12A,ZIC5,CHRNB1,CHRND,DNMT3A,E2F1,EGLN2,EPAS1,F3,FOXA2,GSTM3,HDAC1,IKBKB,KLF14,KSR1,MMP2,NFKB1,PAK2,PIK3CD,SPDEF,TAMALIN,TNFRSF21,TP53* |
| ETF | GVGGMGG | 2,83E+14 | 97/135 | *AHRR,ALX1,BARHL2,BCAR1,BCL6B,CDH13,CDKN2A,CYTL1,DAPK1,EDARADD,EGFR,ERBB2,FGFR3,FZD10,GDNF,GSTP1,HBP1,HLF,HOXA1,HOXA9,HOXB4,HOXD10,HOXD11,IL6,IRX2,ITGA5,LHX1,LRP12,MDK,MEOX2,MLH1,MSX1,NR2E1,OLIG1,ONECUT2,PAX6,PRDM14,RARB,RASSF1,RB1,RUNX1,RXRG,SCT,SFRP1,SHOX2,SIX6,SLIT2,SMAD3,SOX17,TAFA4,TBX5,TCF21,TERT,TFAP2A,TNFRSF25,TRAF1,WT1,ZEB2,ZMIZ1,ZNF577,AKT3,ARRB1,CHD1,CYP1B1,GATA5,GFI1,GNG12,HIVEP3,ITPK1,JAG1,LRP5,MIDEAS,NOS1AP,RARA,SFRP2,ZC3H12A,ZIC5,BST1,CHRNB2,DNMT3A,E2F1,EPAS1,F3,FOXA2,FOXO3,HDAC1,IKBKB,KLF14,KSR1,MMP2,NFKB1,PAK2,PIK3CD,STAT3,TAMALIN,TNFRSF21,TP53* |
| TCF-1 | ACATCGRGRCGCTGW | 3,02E+16 | 101/135 | *AHRR,BARHL2,BCAR1,BCL6B,CDH1,CDH13,CDKN2A,DAPK1,EDARADD,EGFR,ERBB2,F2RL3,FGFR3,FZD10,GDNF,GSTP1,HBP1,HLF,HOXA1,HOXA7,HOXA9,HOXB4,HOXD10,HOXD11,HSPB6,IRX2,ITGA5,KPNA7,LHX1,LRP12,MEIS1,MLH1,MSX1,MYF6,NR2E1,OLIG1,ONECUT2,OSR1,PAX6,PAX8,PAX9,PITX2,PRDM14,RB1,RUNX1,SCT,SFRP1,SHOX2,SIX6,SLIT2,SMAD3,SOX17,TAFA4,TBX5,TERT,TGFBR2,TNFRSF25,TRAF1,WT1,ZEB2,ZMIZ1,AKT3,ARRB1,CHD1,CYP1B1,GATA5,GFI1,GNG12,HIVEP3,ITPK1,JAG1,LRP5,MIDEAS,NOS1AP,PAX5,RARA,SFRP2,ZC3H12A,ZIC5,CHRNB1,CHRNB2,CHRND,DNMT3A,E2F1,EGLN2,EPAS1,F3,FOXA2,FOXO3,GSTM3,IKBKB,KLF14,KSR1,MMP2,NFKB1,PAK2,PIK3CD,STAT3,TAMALIN,TNFRSF21,TP53* |
| TIEG1 | NCCCNSNCCCCGCCCCC | 3,18E+15 | 82/135 | *ALX1,APC,AXL,BARHL2,BCAR1,BCL6B,CDH1,CDKN2A,EGFR,ERBB2,F2RL3,FGFR3,FZD10,GDNF,GSTP1,HBP1,HLF,HOXA1,HOXB4,HOXD11,IRX2,ITGA5,LRP12,MDK,MSX1,ONECUT2,OSR1,PRDM14,PTGER4,RARB,RB1,RUNX1,SCT,SERPINB5,SHOX2,SLIT2,SMAD3,SOX17,TAFA4,TERT,TFAP2A,TGFBR2,TNFRSF25,TRAF1,ZEB2,ZMIZ1,AKT3,ARRB1,CHD1,CYP1B1,GATA5,GFI1,GNG12,ITPK1,JAG1,LRP5,MIDEAS,NOS1AP,RARA,ZC3H12A,ZIC5,BST1,CHRNB1,CHRND,DNMT3A,E2F1,EGLN2,EPAS1,F3,FOXA2,FOXO3,GSTM3,HDAC1,IKBKB,KLF14,KSR1,MMP2,NFKB1,PAK2,PIK3CD,TAMALIN,TP53* |
| MZF-1 | TGGGGAR | 3,43E+16 | 102/135 | *AHRR,APC,AXL,BARHL2,BCL6B,CDH1,CDKN2A,CHRNA1,CXCL8,CYTL1,EDARADD,ERBB2,ESRRG,F2RL3,FGFR3,FZD10,GSTA3,GSTP1,HLF,HOPX,HOXA1,HOXA7,HOXB4,HOXD10,HSPB6,IL1B,IL6,IRX2,ITGA5,KPNA7,LHX1,LMO3,LRP12,MDK,MEIS1,MEOX2,MLH1,MSX1,MYF6,NR2E1,NR3C1,OLIG1,ONECUT2,PAX6,PAX8,PITX2,PRDM14,PTGER4,RARB,RASSF1,RPTOR,RUNX3,RXRG,SERPINB5,SHOX2,SLIT2,SMAD3,SOX17,TCF21,TFAP2A,TNFRSF25,TP63,TRAF1,WT1,ZMIZ1,ZNF154,AKT3,CHD1,FAM98B,GATA5,GFI1,HIVEP3,ITPK1,JAG1,LRP5,MIDEAS,PAX5,RARA,SFRP2,ZC3H12A,ZIC5,BST1,CHRNB2,CHRND,DNMT3A,E2F1,EGLN2,F3,FOXA2,FOXO3,GSTM3,HDAC1,IKBKB,KLF14,MMP2,NFKB1,PAK2,SPDEF,STAT3,TAMALIN,TNFRSF21,TP53* |
| LRF | GGGGKYNNB | 3,61E+15 | 77/135 | *AHRR,AXL,BARHL2,BCAR1,BCL6B,CDH1,CDH13,CDKN2A,CHRNA1,CYTL1,EGFR,ERBB2,ESRRG,F2RL3,FGFR3,FHIT,FZD10,GDNF,GSTP1,HOXA7,HOXB4,HOXD11,HSPB6,IRX2,ITGA5,KPNA7,LRP12,MDK,MEIS1,MEOX2,MSX1,MYF6,ONECUT2,PAX6,PAX9,PRDM14,RASSF1,RB1,RUNX3,SCT,SERPINB5,SLIT2,SMAD3,SOX17,TCF21,TERT,TGFBR2,TNFRSF25,TRAF1,WT1,ZEB2,ZFP42,ZNF154,ARRB1,FAM98B,GFI1,GNG12,ITPK1,JAG1,LRP5,MIDEAS,NOS1AP,RARA,SFRP2,ZC3H12A,ZIC5,CHRNB1,CHRNB2,DNMT3A,F3,FOXA2,HDAC1,KSR1,NFKB1,SPDEF,STAT3,TAMALIN* |
| CKROX | SCCCTCCCC | 3,78E+16 | 75/135 | *AHRR,APC,AXL,BCAR1,CDH1,CDKN2A,ERBB2,F2RL3,FZD10,GDNF,GSTP1,HBP1,HLF,HOPX,HOXA1,HOXB4,HOXD11,HSPB6,IL1B,IRX2,ITGA5,LHX1,LRP12,MDK,MSX1,MYF6,NR2E1,OLIG1,ONECUT2,PAX8,PAX9,PRDM14,RB1,RPTOR,SCT,SHOX2,SIX6,SMAD3,SOX17,TERT,TFAP2A,ZEB2,ZNF577,AKT3,ARRB1,CHD1,CYP1B1,GATA5,GNG12,JAG1,LRP5,MIDEAS,NOS1AP,PAX5,RARA,SFRP2,ZC3H12A,ZIC5,BST1,CHRNB1,CHRNB2,DNMT3A,E2F1,EGLN2,FOXA2,FOXO3,GSTM3,HDAC1,IKBKB,KLF14,KSR1,MMP2,NFKB1,PAK2,PIK3CD* |
| PATZ | GGGGNGGGGGMKGGRRNGGNRN | 4,01E+16 | 83/135 | *ALX1,AXL,BARHL2,BCAR1,BCL6B,CDKN2A,EDARADD,ERBB2,FGFR3,FZD10,GDNF,GSTP1,HBP1,HLF,HOXA1,HOXA7,HOXA9,HOXB4,HOXD11,IL6,IRX2,LRP12,MDK,MSX1,MYF6,NR2E1,OLIG1,ONECUT2,OSR1,PAX9,PRDM14,PTGER4,RB1,RPTOR,RUNX1,SCT,SERPINB5,SHOX2,SIX6,SLIT2,SMAD3,SOX17,TAFA4,TERT,TFAP2A,TRAF1,WT1,ZEB2,AKT3,ARRB1,CHD1,CYP1B1,GATA5,GFI1,GNG12,HIVEP3,ITPK1,JAG1,MIDEAS,NOS1AP,RARA,ZC3H12A,ZIC5,BST1,CHRNB1,CHRNB2,DNMT3A,E2F1,EGLN2,EPAS1,F3,FOXA2,GSTM3,HDAC1,KLF14,KSR1,MMP2,NFKB1,PAK2,PIK3CD,TAMALIN,TNFRSF21,TP53* |
| WT1 | NNGGGNGGGSGN | 4,64E+15 | 69/135 | *APC,AXL,BARHL2,BCAR1,BCL6B,CYTL1,ERBB2,ESRRG,FGFR3,FZD10,GSTA3,GSTP1,HBP1,HLF,HOXA7,HOXB4,HOXD10,HOXD11,HSPB6,IRX2,ITGA5,LHX1,LRP12,MDK,MSX1,MYF6,NR2E1,ONECUT2,OSR1,PAX9,PITX2,PRDM14,RARB,RPTOR,SCT,SIX6,SMAD3,SOX17,TAFA4,TFAP2A,TGFBR2,TNFRSF25,TP63,TRAF1,ZEB2,ZMIZ1,ZNF154,AKT3,ARRB1,CHD1,GFI1,GNG12,JAG1,MIDEAS,RARA,SFRP2,ZIC5,DNMT3A,E2F1,EGLN2,EPAS1,FOXA2,FOXO3,GSTM3,HDAC1,KLF14,MMP2,NFKB1,TAMALIN* |
| RERE | CNGCNSCNNGSRCRGSGSS | 4,95E+15 | 95/135 | *AHRR,AXL,BARHL2,BCAR1,BCL6B,CDH1,CDH13,CYTL1,DAPK1,EDARADD,EGFR,ERBB2,F2RL3,FGFR3,FHIT,FZD10,GDNF,GSTP1,HLF,HOPX,HOXA1,HOXD11,HSPB6,IRX2,LHX1,LRP12,MDK,NR2E1,OLIG1,ONECUT2,OSR1,PAX6,PAX8,PAX9,PITX2,PRDM14,PTGER4,RUNX1,SCT,SERPINB5,SFRP1,SHOX2,SIX6,SLIT2,SMAD3,TAFA4,TCF21,TERT,TFAP2A,TGFBR2,TRAF1,WT1,ZEB2,ZMIZ1,AKT3,ARRB1,CHD1,CYP1B1,GATA5,GFI1,GNG12,HIVEP3,ITPK1,JAG1,LRP5,MIDEAS,NOS1AP,PAX5,RARA,SFRP2,ZC3H12A,ZIC5,BST1,CHRNB1,CHRNB2,CHRND,DNMT3A,E2F1,EGLN2,EPAS1,F3,FOXO3,GSTM3,HDAC1,IKBKB,KLF14,KSR1,MMP2,NFKB1,PAK2,PIK3CD,SPDEF,TAMALIN,TNFRSF21,TP53* |
